# Genomewide Analysis of PRC1 and PRC2 Occupancy Identifies Two Classes of Bivalent Domains

**DOI:** 10.1371/journal.pgen.1000242

**Published:** 2008-10-31

**Authors:** Manching Ku, Richard P. Koche, Esther Rheinbay, Eric M. Mendenhall, Mitsuhiro Endoh, Tarjei S. Mikkelsen, Aviva Presser, Chad Nusbaum, Xiaohui Xie, Andrew S. Chi, Mazhar Adli, Simon Kasif, Leon M. Ptaszek, Chad A. Cowan, Eric S. Lander, Haruhiko Koseki, Bradley E. Bernstein

**Affiliations:** 1 Molecular Pathology Unit and Center for Cancer Research, Massachusetts General Hospital, Charlestown, Massachusetts, United States of America; 2 Department of Pathology, Harvard Medical School, Boston, Massachusetts, United States of America; 3 Broad Institute of Harvard and MIT, Cambridge, Massachusetts, United States of America; 4 Division of Health Sciences and Technology, MIT, Cambridge, Massachusetts, United States of America; 5 Bioinformatics Program and Department of Biomedical Engineering, Boston University, Boston, Massachusetts, United States of America; 6 RIKEN Research Center for Allergy and Immunology, Tsurumi-ku, Yokohama, Japan; 7 School of Engineering and Applied Sciences, Harvard University, Cambridge, Massachusetts, United States of America; 8 Department of Computer Science, University of California Irvine, Irvine, California, United States of America; 9 Cardiology Division, Massachusetts General Hospital, Boston, Massachusetts, United States of America; 10 Harvard Stem Cell Institute, Cambridge, Massachusetts, United States of America; 11 Stowers Medical Institute, Center for Regenerative Medicine, Cardiovascular Research Center, Massachusetts General Hospital, Boston, Massachusetts, United States of America; 12 Whitehead Institute for Biomedical Research, MIT, Cambridge, Massachusetts, United States of America; Netherlands Cancer Institute, The Netherlands

## Abstract

In embryonic stem (ES) cells, bivalent chromatin domains with overlapping repressive (H3 lysine 27 tri-methylation) and activating (H3 lysine 4 tri-methylation) histone modifications mark the promoters of more than 2,000 genes. To gain insight into the structure and function of bivalent domains, we mapped key histone modifications and subunits of Polycomb-repressive complexes 1 and 2 (PRC1 and PRC2) genomewide in human and mouse ES cells by chromatin immunoprecipitation, followed by ultra high-throughput sequencing. We find that bivalent domains can be segregated into two classes—the first occupied by both PRC2 and PRC1 (PRC1-positive) and the second specifically bound by PRC2 (PRC2-only). PRC1-positive bivalent domains appear functionally distinct as they more efficiently retain lysine 27 tri-methylation upon differentiation, show stringent conservation of chromatin state, and associate with an overwhelming number of developmental regulator gene promoters. We also used computational genomics to search for sequence determinants of Polycomb binding. This analysis revealed that the genomewide locations of PRC2 and PRC1 can be largely predicted from the locations, sizes, and underlying motif contents of CpG islands. We propose that large CpG islands depleted of activating motifs confer epigenetic memory by recruiting the full repertoire of Polycomb complexes in pluripotent cells.

## Author Summary

Polycomb-group (PcG) proteins play essential roles in the epigenetic regulation of gene expression during development. PcG proteins are repressors that catalyze lysine 27 tri-methylation on histone H3. They are antagonized by trithorax-group proteins that catalyze lysine 4 tri-methylation. Recent studies of ES cells revealed a novel chromatin pattern consisting of overlapping lysine 27 and lysine 4 tri-methylation. Genomic regions with these opposing modifications were termed “bivalent domains” and proposed to silence developmental regulators while keeping them “poised” for alternate fates. However, our understanding of PcG regulation and bivalent domains remains limited. For instance, bivalent domains affect over 2,000 promoters with diverse functions, which suggests that they may function in diverse cellular processes. Moreover, the mechanisms that underlie the targeting of PcG complexes to specific genomic regions remain completely unknown. To gain insight into these issues, we used ultra high-throughput sequencing to map PcG complexes and related modifications genomewide in human and mouse ES cells. The data identify two classes of bivalent domains with distinct regulatory properties. They also reveal striking relationships between genome sequence and chromatin state that suggest a prominent role for the DNA sequence in dictating the genomewide localization of PcG complexes and, consequently, bivalent domains in ES cells.

## Introduction

Increasing evidence suggests that Polycomb- (PcG) and trithorax-group (trxG) proteins and associated histone modifications are critical for the plasticity of the pluripotent state, for the dynamic changes in gene expression that accompany ES cell differentiation, and for subsequent maintenance of lineage-specific gene expression programs [1]–[4].

PcG proteins are transcriptional repressors that function by modulating chromatin structure [2]–[4]. They reside in two main complexes, termed Polycomb repressive complexes 1 and 2 (PRC1 and PRC2). PRC2 contains Ezh2, which catalyzes histone H3 lysine 27 tri-methylation (H3K27me3), as well as Eed and Suz12. PRC1 contains Ring1, an E3 ubiquitin ligase that mono-ubiquitinylates histone H2A at lysine 119 (H2Aub1) [5],[6]. Other PRC1 components include Bmi1, Mel-18, and Cbx family proteins with affinity for H3K27me3 [2],[3].

Interplay between PcG complexes and modified histones has been proposed to mediate stable transcriptional repression [2],[3]. In the prevailing model, PRC2 is recruited to specific genomic locations where it catalyzes H3K27me3. The modified histones in turn recruit PRC1, which catalyzes H2Aub1 and thereby impedes RNA polymerase II elongation [7],[8]. PRC1 may also affect PRC2 function through as yet undefined mechanisms [2],[3].

Several groups have combined chromatin immunoprecipitation (ChIP) with microarrays to examine the genomic localizations of individual PcG subunits [9]–[13]. Lee et al used tiling arrays to map the PRC2 subunit Suz12 in human ES cells, identifying nearly 2000 gene targets. Boyer et al used promoter arrays to identify 512 genes co-occupied by PRC2 and PRC1 components in mouse ES cells. In both studies, the implicated gene sets were highly enriched for developmental transcription factors (TFs), many of which become de-repressed upon ES cell differentiation or in a PRC2-deficient background.

Concurrent studies of histone methylation in ES cells led to the unexpected finding that virtually all sites of PcG activity not only carry the repressive H3K27me3 modification, but are also strongly enriched for the activating, trxG-associated H3 lysine 4 tri-methylation (H3K4me3) mark [14],[15]. Genomic regions with the two opposing modifications were termed ‘bivalent domains’ and proposed to silence developmental regulators while keeping them ‘poised’ for alternate fates. Upon ES cell differentiation, most bivalent promoters resolve to a ‘univalent’ state. Induced genes become further enriched for H3K4me3 and lose H3K27me3, while many non-induced genes retain H3K27me3 but lose H3K4me3 [15],[16].

Despite this progress, our understanding of PcG regulation and bivalent domains remains limited. In the current study we sought to address two outstanding issues. The first relates to whether all bivalent domains have the same regulatory structure. The recent observation that human and mouse ES cells show overlapping H3K27me3 and H3K4me3 at over 2000 promoters, only a portion of which have developmental functions, suggests that bivalent domains may reflect multiple, distinct regulatory entities [16]–[18]. The second relates to the mechanisms that underlie the targeting of PcG complexes and the establishment of bivalent domains in ES cells. In *Drosophila*, PcG complexes are recruited to DNA elements termed Polycomb response elements (PREs). However, mammalian equivalents of these elements have yet to be identified [4].

We addressed these outstanding issues through genomewide analysis of PcG complex localization in mouse and human ES cells. We used the newly developed ‘ChIP-Seq’ method, which leverages ultra high-throughput sequencing to generate uniquely comprehensive maps of protein-DNA interactions [16],[19].

The data reveal two classes of bivalent domains with distinct regulatory properties. The first class corresponds to bivalent domains with both PRC2 and PRC1. These ‘PRC1-positive’ bivalent domains show striking evolutionary conservation, correspond to large H3K27me3 regions in ES cells that are significantly more likely to retain H3K27me3 upon differentiation, and account for a vast majority of implicated developmental regulator genes. By contrast, PRC1-negative bivalent domains, which are exclusively bound by PRC2, are weakly conserved, poorly retain H3K27me3, and largely correspond to membrane proteins or genes with unknown functions. Remarkably, computational genomic analysis of the ChIP-Seq data suggests a simple genomic code in which the locations, sizes and motif contents of CpG islands may predict the genomewide localizations of PRC2, PRC1 and bivalent domains in ES cells. Based on these data, we propose a model in which large CpG islands depleted of activating transcription factor motifs confer epigenetic memory elements through mammalian development by recruiting PRC2 and PRC1 during early embryogenesis.

## Results

### Overview of ChIP-Seq Datasets

To gain insight into the structure, function and conservation of bivalent chromatin, we used ChIP-Seq to acquire genomewide maps of PcG complex components and related histone modifications in ES cells (Table S1). Chromatin from mouse v6.5 ES cells or human H9 ES cells was immunoprecipitated using antibodies against Ezh2, Suz12, Ring1B, H3K4me3, H3K27me3 or H3K36me3 (Materials and Methods). We also used biotin-streptavidin interaction (bioChIP) to purify chromatin from a transgenic mouse ES line in which endogenous Ring1B is fused to biotin ligase recognition peptide. DNA isolated in each ChIP experiment was sequenced to high depth using the Illumina Genome Analyzer. Aligned reads were integrated into maps that indicate enrichment of a given epitope as a function of genome position. In total, we created eight genomewide maps that each reflects two to eleven million aligned reads and together represent over 2 Gb of sequence. All data are publicly available at http://www.broad.mit.edu/seq_platform/chip/.

### Evolutionary Conservation of Chromatin State in ES Cells

**Figure 1:**
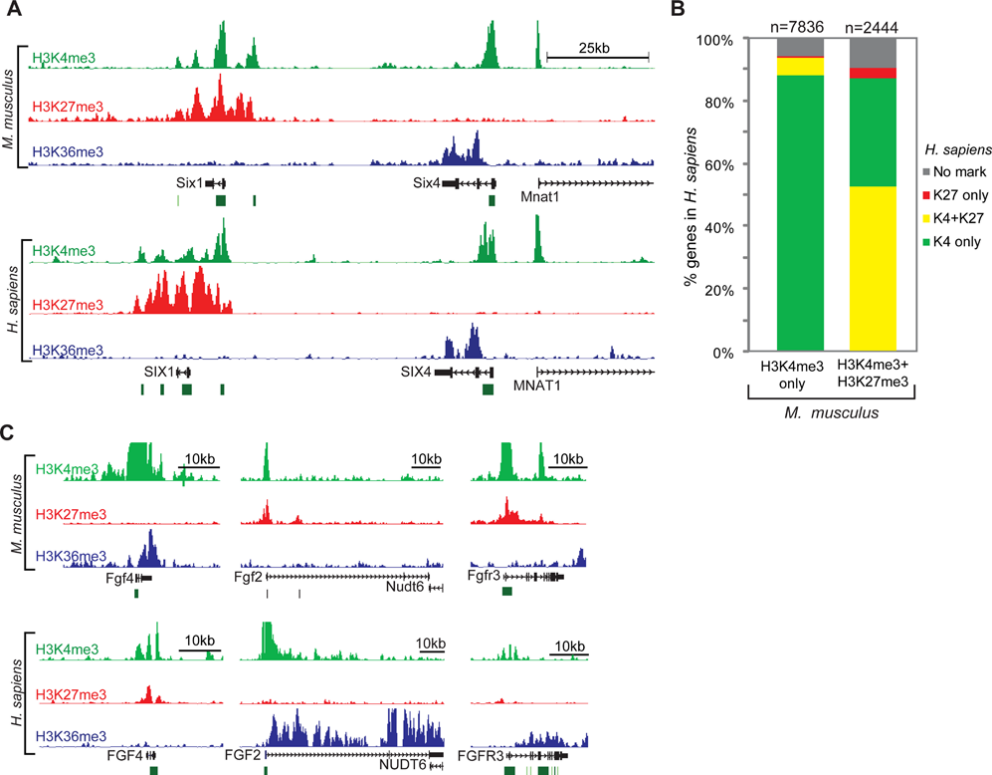
Conservation of chromatin state in mouse and human ES cells. (A) ChIP-Seq signals for H3K4me3 (green), H3K27me3 (red) and H3K36me3 (blue) are plotted across 120 kb of orthologous sequence in mouse and human ES cells. (B) The proportion of promoters that have a given chromatin state in human ES cells is indicated contingent on their state in mouse ES cells. (C) ChIP-Seq signals are shown for developmental regulator loci with divergent chromatin state in mouse and human ES cells. The divergent states correspond to known differences between the two pluripotency models (see text).

The availability of genomewide data for mouse and human ES cells acquired using identical antibodies and methodologies provides an opportunity to study the conservation of chromatin state in pluripotent cells. We systematically compared chromatin state at 13,200 orthologous promoters, identifying striking similarities at orthologous genomic loci (Figure 1A, Figure S1; Table S2, S3, and S4).

In both mouse and human ES cells, roughly three-quarters of gene promoters are marked by H3K4me3. There is strong correspondence between species as >94% of promoters with H3K4me3 in mouse also carry H3K4me3 in human. Roughly one fifth of H3K4me3 promoters also carry H3K27me3, and thus are bivalent (mouse: n = 2978; human: n = 2529) (Figure S1C). There is again strong conservation, with more than half of bivalent mouse promoters also carrying bivalent chromatin in human ES cells (Figure 1B and Figure S1A). As shown previously, many bivalent mouse promoters correspond to homeobox TFs or other developmental regulators [14],[15]. These gene categories show particularly strong conservation of chromatin state, with roughly 70% correspondence between mouse and human. Still, there are numerous developmental regulators whose chromatin state differs between species (Figure S3). Closer inspection of these genes reveals a number of interesting cases that appear to reflect biological differences between the two pluripotency models:

1. The promoters of Fgf2, Fgfr3, Activin A, Lefty1 and Lefty2 are bivalent in mouse ES cells but show active ‘H3K4me3 only’ states in human (Figure 1C). This is consistent with known expression patterns for these genes, which are associated with the human ES cell-specific Activin/NODAL pathway [20]–[22]. Another example is SOCS1, an inhibitor of STAT3 signaling that is specifically expressed in human ES cells where it may block response to LIF [23].
2. Conversely, the chromatin maps reveal developmental regulators that are bivalent only in human ES cells, and these may also relate to known physiologic differences between the models (Figure 1C). Examples include Fgf4 and Gbx2, which are associated with the inner cell mass and specifically expressed in mouse ES cells [20],[24],[25].

Thus, comparative analysis of human and mouse ES cells suggests extensive conservation of the pluripotent chromatin state while also illuminating divergent chromatin regulation associated with signaling pathways and transcriptional programs known to vary between the studied cell models (see also Figure S3). The strong conservation of bivalent domains seen here contrasts with the surprisingly weak correspondence observed previously for Oct4 and Nanog targets between mouse and human ES cells [26]. Consistent with prior studies, our data suggest that global patterns of H3K27me3 and H3K4me3 are intimately tied to transcriptional programs and cellular state, and that the bivalent combination is a conserved mark of silent developmental regulators in pluripotent cells.

### PcG Complex Occupancy Defines Two Classes of Bivalent Domains

#### PRC2 Occupies Essentially All Bivalent Domains

**Figure 2:**
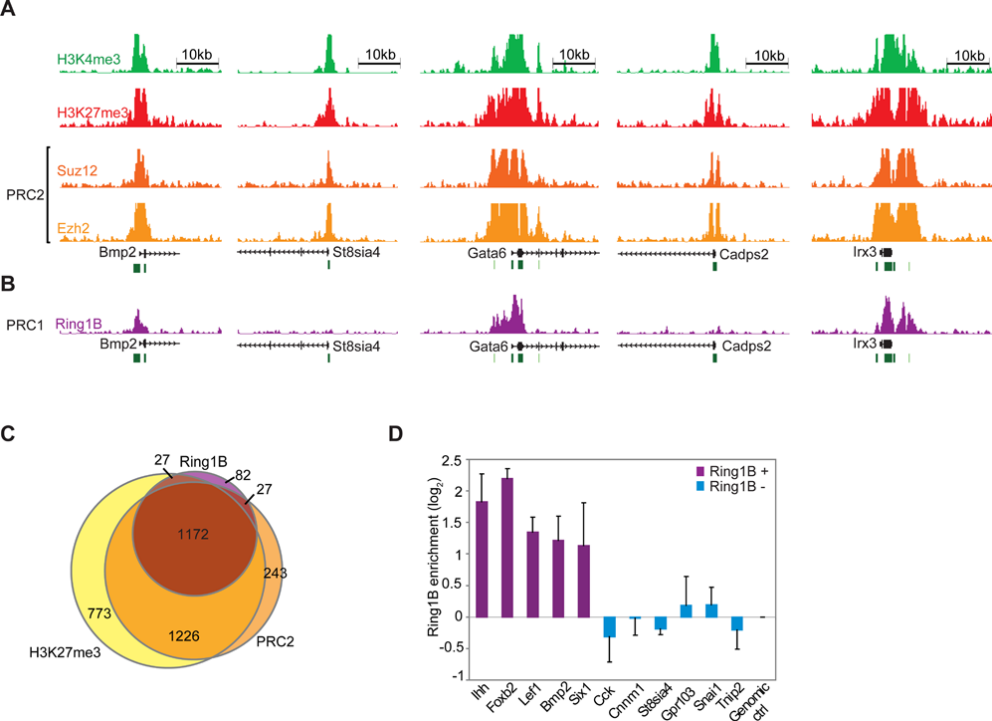
PcG complex occupancy at bivalent domains. (A) ChIP-Seq signals are shown for H3K4me3, H3K27me3 and PRC2 subunits, Suz12 and Ezh2, at a representative panel of bivalent gene promoters. (B) ChIP-Seq signal for the PRC1 subunit Ring1B at these loci. (C) Venn diagram illustrating overlap between promoters marked by H3K27me3, PRC2 and Ring1B. (D) ChIP-qPCR data for Ring1B at bivalent promoters classified by ChIP-Seq as ‘Ring1B-positive’ or ‘Ring1B-negative’. Error bars show standard deviation.

To gain insight into the establishment and function of bivalent domains, we next considered the localization of PcG complexes in mouse ES cells. ChIP-Seq maps for the PRC2-components Ezh2 and Suz12 reveal >3000 sites in the mouse genome significantly enriched for one or both factors. Roughly three-quarters of these PRC2 bound sites correspond to known gene promoters: Ezh2 occupies 2461 promoters, while Suz12 occupies 1944 promoters. There is extensive overlap between these sets of promoters, with more than 89% of Suz12 targets also having Ezh2 (r_phi_ = 0.77). There is also overwhelming overlap with bivalent promoters: nearly all Suz12 and Ezh2 targets have bivalent histone markings and, conversely, 78% of bivalent promoters have Ezh2 or Suz12 (Figure 2A,C).

Since PRC2 is the only known complex capable of catalyzing H3K27me3 [2], we considered the minority (22%) of bivalent promoters for which PRC2 was not detected by ChIP-Seq. Many of these promoters show relatively low levels of H3K27me3, and we considered whether PRC2 was simply missed due to sensitivity or thresholding issues. Consistent with this possibility, ChIP with quantitative real-time PCR (qPCR) confirmed modest but significant Ezh2 enrichment at each of these promoters (ratios from 2- to 7-fold; Figure S2A). This suggests that PRC2 is present at essentially all bivalent promoters. Notably, the correspondence between H3K27me3 and PRC2 is not limited to annotated gene promoters, as near-universal PRC2 binding is also evident at the roughly 1000 sites of bivalent chromatin that do not correspond to known genes (see Materials and Methods).

#### PRC1 Occupies a Conserved Subset of Bivalent Domains

We next turned to examine PRC1 localization, focusing on its catalytic component Ring1B. ChIP-Seq maps reveal roughly 1500 significantly enriched genomic sites in mouse ES cells, including 1308 annotated gene promoters. Nearly all (90%) Ring1B targets correspond to bivalent, PRC2-bound genomic regions. However, just 39% of bivalent promoters are enriched for Ring1B (Figure 2B,C). This occupancy rate is roughly half that observed for Ezh2. As an added measure, we created an Ezh2 ChIP-Seq dataset with exactly the same number of reads as the Ring1B dataset (by randomly selecting reads). Analysis of this truncated dataset reveals Ezh2 binding at 74% of bivalent promoters (compare to 75% for the full Ezh2 ChIP-Seq dataset). Hence, sequencing depth does not account for the difference between Ezh2 and Ring1B occupancy.

Thus, ChIP-Seq analysis suggests that while PRC2 is ubiquitously present at bivalent promoters, PRC1 occupies only a distinct subset. Since PRC2 and PRC1 have generally been described at common genes and loci [9],[10], we sought to confirm this unexpected result by orthogonal approaches, as follows:

1. First, we used ChIP and qPCR to exclude the possibility that the absence of Ring1B at a subset of bivalent promoters reflected a lack of sensitivity of the ChIP-Seq data. This analysis confirmed that Ring1B-negative bivalent promoters also do not show any enrichment by qPCR (Figure 2D).
2. Next, to rule out antibody-related bias, we used bioChIP to purify Ring1B-bound chromatin from transgenic ES cells carrying a fusion between Ring1B and biotin ligase recognition peptide (Figure S2B). Ring1B-positive bivalent promoters again showed consistent enrichment, while Ring1B-negative bivalent promoters showed similar enrichment to background controls.
3. Third, to test whether the existence of Ring1B-positive and negative bivalent domains is a conserved phenomenon, we examined Ring1B occupancy in human ES cells by ChIP-Seq. We again found that Ring1B occupies only a subset of bivalent domains. The locations of PRC1 show remarkable cross-species conservation: 60% of Ring1B-positive promoters in human are also Ring1B-positive in mouse (Table S4).
4. Finally, to confirm that Ring1B status is reflective of PRC1 status, we studied the localization of a distinct PRC1 component, Bmi1. Using an epitope tagged construct in ES cells, we showed that Bmi1 specifically localizes to Ring1B-positive bivalent domains (Figure S2C). This suggests that our findings on Ring1B generally apply to the PRC1 complex. Henceforth, the two sets of bivalent domains are notated as ‘PRC1-positive’ and ‘PRC1-negative’.

### PRC1-Bound Bivalent Domains Are Functionally Distinct

The identification of a distinct set of bivalent promoters targeted by Ring1B prompted us to investigate the functional significance of PRC1 occupancy. We made several striking observations relevant to chromatin regulation, epigenetic memory, development and differentiation:

#### PRC1 Occupancy Correlates with Functional Repression

**Figure 3:**
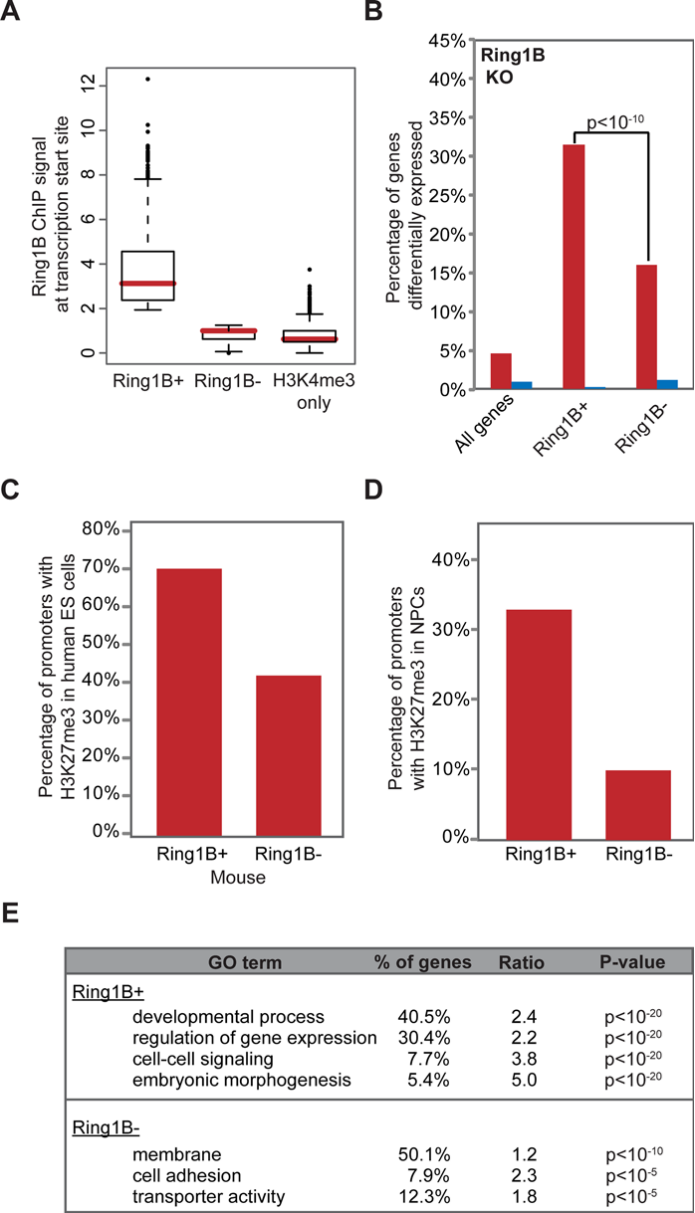
PRC1-positive bivalent domains are functionally distinct. (A) Box plot shows 25^th^, 50^th^ and 75^th^ percentile Ring1B ChIP-Seq signals for Ring1B-positive bivalent promoters, Ring1B-negative bivalent promoters, and for H3K4me3 only promoters. (B) Plot illustrates fraction of genes up-regulated (red) or down-regulated (blue) in PRC1-deficient ES cells for the indicated gene sets (see text for details on Ring1A/B dKO ES cell model). De-repression is evident for a significantly greater proportion of PRC1-positive bivalent promoters (p-value by Fisher's exact test). (C) The proportion of bivalent mouse promoters for which the human ortholog also carries H3K27me3 is indicated, contingent on Ring1B status in mouse ES cells. (D) The proportion of bivalent promoters for which H3K27me3 is retained in ES cell-derived neural progenitors (‘NPCs’), contingent on Ring1B status in mouse ES cells. (E) Gene Ontology categories over-represented in PRC1-positive or PRC1-negative bivalent gene sets.

We first considered whether physical targets of PRC1, as defined above, are also regulated by the complex. Since Ring1B and Ring1A are functionally redundant, we employed a conditional Ring1A/B double-knockout ES cell system in which Ring1B depletion is induced by addition of 4-hydroxy tamoxifen (OHT) [13]. We profiled expression changes after 48 hours of OHT treatment, at which time Ring1B protein levels are markedly depleted while Oct4 levels remain essentially unchanged [8],[13]. We found that 32% of PRC1-positive bivalent promoters are up-regulated by at least 50%, compared to just 5% of all genes (Figure 3B). A much smaller proportion of PRC1-negative bivalent promoters are up-regulated at this time point (16%). The difference between the two sets is statistically significant (p<10^−10^), and is not explained by baseline expression levels as bivalent promoters show very low activity, regardless of PRC1 status.

Several factors could contribute to de-repression of this smaller set of PRC1-negative bivalent promoters. The changes may reflect indirect effects as expression is measured after 2 days of OHT treatment. Also, the Ring1 knockout experiment and the location analyses were done in different ES lines, and this could be the basis of some of the discrepancy. Nonetheless, the fact that the PRC1-positive set shows a significantly greater response indicates that PRC1 occupancy correlates with functional repression. As a control, we examined expression changes associated with PRC2 loss. We found that PRC1-positive and PRC1-negative bivalent promoters are de-repressed to roughly equal extents in ES cells lacking the PRC2 component Eed (Figure S4) [13].

#### PRC1-Positive Bivalent Domains Correspond to Large and Conserved Sites of H3K27me3

Next, we asked whether the patterns of histone modification vary between the two sets of bivalent domains. We observed two significant trends. First, PRC1-positive bivalent domains are associated with much larger regions of H3K27me3 than PRC1-negative bivalent domains (median size of 3.2 kb versus 1.0 kb). The large size is consistent with a proposed role for H3K27me3 in PRC1 recruitment [2],[3]. Second, PRC1-positive bivalent domains exhibit greater conservation of chromatin state: bivalent mouse promoters with PRC1 have a bivalent human ortholog in 71% of cases, compared to just 43% of bivalent mouse promoters without PRC1 (p<10^−10^; Figure 3C). Thus, PRC1 occupancy correlates with larger bivalent domains that appear to reflect highly conserved functions.

#### PRC1-Positive Bivalent Domains Correspond to Developmental Regulator Genes

Next, we examined the gene targets associated with the different classes of bivalent promoters. The PRC1-positive set contains a dramatic enrichment of genes encoding TFs (30%, p<10^−20^), including members of the Hox, Sox, Pax and Pou domain families, or cell signaling and morphogenesis molecules, such as Wnts and Fgfs (Figure S3). In contrast, the PRC1-negative set of bivalent promoters is instead over-represented for genes that encode membrane proteins (50%; p<10^−10^). Remarkably, despite the strong correlation of PcG proteins with developmental TFs, this PRC1-negative (PRC2-only) subset of bivalent domains shows statistically significant depletion of TF genes relative to the genome average (4.1% vs 10.2%; p<10^−10^ ).

#### PRC1-Positive Bivalent Domains Efficiently Maintain Repressive Chromatin Environment

Finally, we compared the behavior of PRC1-positive and PRC1-negative bivalent promoters upon ES cell differentiation. We examined ChIP-Seq data for a population of neural progenitors (NPCs) derived from the same ES cell line [16]. Since PRC1 is implicated in the maintenance of a repressive chromatin state, we reasoned that promoters with PRC1 should more efficiently retain H3K27me3 upon differentiation. Consistent with this hypothesis, we found that 33% of PRC1-positive bivalent promoters retain H3K27me3 in the NPCs, compared to just 10% of PRC1-negative bivalent promoters (p<10^−10^) (Figure 3D). Many PRC1-positive bivalent promoters that lose the repressive mark upon differentiation do so in association with transcriptional activation as roughly one-fifth are induced at least 5-fold in the NPCs. Thus, PRC1 occupancy is associated with more stable retention of PcG-associated chromatin marks through differentiation.

We conclude that two distinct sets of bivalent domains can be defined based on PcG complex occupancy in ES cells. Bivalent domains that carry both PRC2 and PRC1 are larger, more conserved and more efficiently retained through differentiation. They account for the vast majority of implicated developmental regulators. By contrast, bivalent domains occupied by PRC2 only are poorly maintained, correspond to distinct non-developmental gene sets, and thus may reflect alternate regulatory processes.

### Sequence Elements and Motifs Predict PcG Complex Localization in ES Cells

We next studied the chromatin maps to gain insight into another fundamental unanswered question – namely, the mechanisms that underlie the initial recruitment of PcG complexes and the formation of bivalent domains in ES cells. The extensive epigenetic reprogramming that precedes the pluripotent state suggests that elements in the genomic sequence itself must play central roles in this process [1],[27],[28]. Yet the identity of these PcG-determining sequence elements has remained elusive.

#### PRC2 Associates with CG-Rich Sequences Genomewide

To identify sequence elements that could contribute to PcG recruitment, we applied computational sequence analysis and the new ChIP-Seq data. We focused initially on Ezh2, reasoning that this catalytic PRC2 subunit would most closely reflect the initial recruitment mechanisms. Bivalent domains and PcG target sites have been shown previously to correlate with CG-rich DNA; for example, ∼50% of Suz12 binding sites in human ES cells correspond to CpG islands [11],[16],[29]. The ChIP-Seq data for mouse Ezh2 reveal an even higher correspondence, with a full 88% of enriched intervals coinciding with an annotated CpG island. H3K27me3-enriched intervals similarly correlate with CpG islands in 79% of cases. Remarkably, the fraction of Ezh2/H3K27me3 sites that coincide with CpG islands is substantially higher than that of H3K4me3 (68%), which has previously been associated with CpG islands [15]. It is also far greater than that of other chromatin structures (Figure S5), including H3K9me3 (1.1%) and H4K20me3 (0.7%).

When we examined the small minority (12%) of Ezh2 binding sites that do not correspond to an annotated CpG island, we found that three-quarters of these sites overlap highly CG-rich sequences that just fall short of the defined threshold for CpG islands (see Materials and Methods). Including those sites, >97% of Ezh2 binding sites in the ES cell genome correspond to annotated CpG islands or other highly CG-rich sequences. These results suggest that such CG-rich sequences, known to be largely un-methylated at the DNA level in ES cells [27], may contribute to the recruitment of PRC2 and the subsequent establishment of H3K27me3 at bivalent domains.

**Figure 4:**
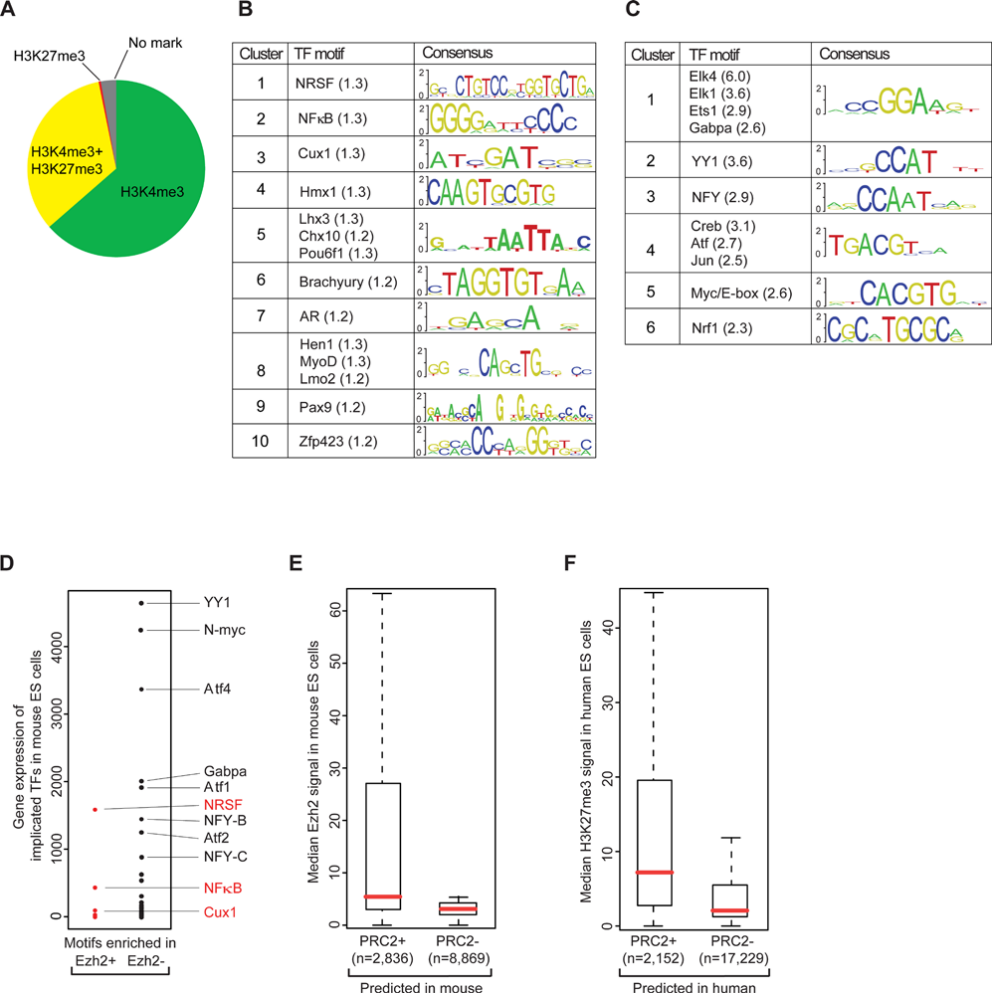
CG-density and DNA motif occurrences predict genomewide PcG complex localization. (A) Proportion of CpG islands with a given chromatin state in mouse ES cells. More than 97% of Ezh2 sites in mouse ES cells correspond to CpG islands or other highly CG-rich sequences. A systematic screen reveals sets of DNA motifs over-represented in (B) Ezh2-positive CpG islands or (C) Ezh2-negative CpG islands (enrichment in parentheses). (D) Expression levels of implicated TFs in mouse ES cells. Motifs enriched in Ezh2-positive CpG islands correspond to repressors or to TFs that are not expressed. Motifs enriched in Ezh2-negative CpG islands correspond to highly expressed activators. (E) Ezh2 ChIP-Seq signals for CpG islands predicted as PRC2-positive or PRC2-negative based on motif occurrences. (F) H3K27me3 ChIP-Seq signals for human ES cells for CpG islands predicted to be PRC2-positive or PRC2-negative based on occurrences of the motifs originally identified in mouse.

Still, only a minority of CpG islands carries Ezh2 or H3K27me3 in ES cells – that is, are PRC2-positive. Most are enriched for H3K4me3 only and are PRC2-negative (Figure 4A). We thus considered whether additional sequence characteristics distinguish between PRC2-positive and PRC2-negative CpG islands. We collated two sets of CpG islands, one showing clear Ezh2 binding based on ChIP-Seq (n = 2608) and the other lacking any Ezh2 signal (n = 9097). To maximize the power of our analysis, we excluded a subset of CpG islands showing intermediate levels of Ezh2 enrichment (n = 3443).

We considered CpG island length, CG density and the frequency of all possible dinucleotides (Figure S6) as potential characteristics. PRC2-positive CpG islands show a greater median length (721 bp vs 526 bp) and a slightly lower median CpG observed-to-expected ratio (0.88 vs 0.92). However, the overall distributions of length and ratio are largely similar and do not discriminate between PRC2-positive and negative sets.

We also compared the conservation properties of these CpG island sets. Mammalian genomes contain ∼200 large regions characterized by striking enrichment for highly conserved non-coding elements [30],[31] and exceptionally low CpG divergence rates [32]. These loci contain promoters for many developmental genes, most of which are bivalent in ES cells [33]. Although it has been suggested that conserved elements within these loci contribute to PcG recruitment, we find that only ∼10% of Ezh2 binding sites occur within these regions. Overall, we find that PRC2-positive CpG islands show modestly higher sequence conservation relative to PRC2-negative islands, but with overlapping distributions (Materials and Methods). Thus, conservation analysis does not present an obvious explanation for observed PRC2 binding patterns.

#### PRC2-Positive CpG Islands Can Be Distinguished Based on Motif Content

Because the distinction between PRC2-positive and PRC2-negative CpG islands is not explained by simple sequence composition, we next considered more complex sequence motifs. In *D. melanogaster*, PcG recruitment is mediated by combinations of motifs recognized by specific TFs [4]. We thus explored whether TF motifs could predict PRC2 localization in mammalian ES cells. Since the motifs and TFs implicated in fly show little or no conservation in vertebrates, we broadened our analysis to include all 668 vertebrate DNA binding motifs annotated in the TRANSFAC and Jaspar databases [34],[35].

We used the MAST algorithm [36] and position weight matrices (PWMs) from these databases to identify motifs. Taking an unbiased approach, we searched for motifs over-represented in either Ezh2-positive or Ezh2-negative CpG islands. Over-represented motifs were ranked by enrichment ratio, and their significance was confirmed using Fisher's exact test. We also excluded the possibility that enriched motifs simply reflected differences in underlying nucleotide content by repeating each survey with scrambled PWMs. Finally, since there is redundancy among factors and PWMs in the TRANSFAC and Jaspar databases, a clustering algorithm was used to collapse highly similar PWMs to a single representative motif. This analysis yielded a total of 14 motifs enriched between 1.2 and 1.3-fold in the Ezh2-positive CpG islands, and these fall into 10 motif clusters. It also revealed 11 motifs enriched between 2.3 and 6.0-fold in the Ezh2-negative CpG islands, falling into 6 clusters (Figure 4B,C, Figure S8).

We initially focused on the motifs associated with Ezh2-positive CpG islands as these could potentially mediate PRC2 recruitment. Although the enrichment ratios were relatively low, it is conceivable that combinations of factors might be required, as in *Drosophila*. However, most of the corresponding TFs are not actually expressed in ES cells, but rather are expressed in differentiated cells. These include developmental regulators induced along specific differentiation pathways, such as MyoD (myogenesis), Lmo2 (hematopoiesis), Brachyury (paraxial mesoderm) and Pou6F1 (neurogenesis) [37]–[40]. PRC2 targets include many developmental genes with complex expression patterns which may explain why they are enriched for lineage-specifying TF motifs. Hence, it is unlikely that these non-expressed TFs contribute to PRC2 localization in ES cells.

However, three of the factors identified in the Ezh2-positive islands are expressed in ES cells, and these cases are illustrative (Figure 4D). The most highly-expressed is neuron-restrictive silencing factor (NRSF/REST), a potent transcriptional repressor essential for ES cell pluripotency [41]. Notably, the NRSF motif is among the best characterized and highly predictive binding elements in mammalian genomes [42]. A second expressed factor is Cux1, which also functions as a transcriptional repressor [43]. The third expressed factor is NFκB, a widely studied transcriptional regulator with diverse functions related to immunity, inflammation and differentiation [44]. Although NFκB is clearly expressed, its activity is strongly inhibited in ES cells by the pluripotency factor Nanog [45]. Thus, motifs enriched in Ezh2-positive CpG islands are recognized either by repressors or by TFs that are inactive in ES cells (see Text S2).

Next, we turned to examine motifs enriched in the Ezh2-negative CpG islands. We were immediately struck that these motifs are recognized by several well-characterized classes of transcriptional activators that are highly expressed in ES cells (Figure 4C,D). Some of the implicated factors have key functions in the ES cell regulatory network (e.g., NFY, Myc) while others are constitutive activators with general housekeeping functions (e.g., Ets1; see Text S2) [46]–[48]. The magnitudes of enrichment observed for these activating motifs are much greater than those observed for motifs identified in Ezh2-positive sequences above. Thus, the strongest sequence correlate of Ezh2 binding at a CpG island appears to be the absence of motifs capable of conferring transcriptional activity.

A simple count of the motif occurrences within a CpG island allows accurate prediction of roughly two-thirds of Ezh2 binding sites (see Materials and Methods; Figure 4E). This compares favorably with the Polycomb response elements predicted in *Drosophila*, which are present at 6 to 27% of experimentally-determined PcG binding sites [4], [49]–[51]. Notably, the motif occurrences we identified in mouse also have considerable predictive value for identifying PcG targets in human ES cells (Figure 4F).

In sum, we find that PRC2-positive CpG islands are characterized by an over-representation of repressor motifs and a strong depletion of transcriptional activator motifs. While it is possible that the implicated repressors directly mediate PRC2 recruitment, each has been well-studied and linked to distinct biological processes. Rather, we favor the view that the paucity of activating motifs and, to a lesser extent, the presence of repressive motifs dictate a transcriptionally inactive state in ES cells that is permissive to PRC2 binding. We suggest that CpG islands play a central role in PRC2 recruitment and, in the absence of transcriptional activity, assume a bivalent chromatin state by ‘default’ in ES cells (see Discussion).

#### PRC1 Occupies Large PRC2-Positive CpG Islands

Lastly, we considered whether PRC1 association can also be predicted from genome sequence. PRC1 occupies roughly half of all PRC2 sites in ES cells, and is essentially never observed in the absence of this second PcG complex. We collated and compared two sets of Ezh2-positive CpG islands, one with Ring1B (n = 1036) and the other without Ring1B (n = 981) (see Methods). We found no significant differences in nucleotide content (CG-density, dinucleotide frequencies) or in the occurrences of the motifs discussed above.

Rather, the best predictor appears to be the length of CG-rich DNA. PRC1-positive CpG islands are roughly twice as large as those that carry only PRC2 (Figure S9). They are also much more likely to reside in close proximity to other bivalent CpG islands. Consideration of CpG island size and proximity to other bivalent islands enables accurate prediction of PRC1 status for >70% of PRC2-positive CpG islands (see Materials and Methods). Thus, our findings suggest that the genomewide localization of the two main PcG complexes in ES cells may be largely predicted from the location, size and underlying motif content of CpG islands.

## Discussion

We have applied ChIP-Seq and computational genomic analysis to study the genomewide distributions of key histone modifications and PcG subunits in mouse and human ES cells, thereby gaining insight into the structure, function and establishment of bivalent domains.

The ChIP-Seq data reveal two distinct sets of bivalent domains in ES cells. One set, defined based on co-occupancy by both PRC1 and PRC2, shows special epigenetic properties, including higher evolutionary conservation of chromatin state and robust retention of repressive chromatin through differentiation. This set is exquisitely enriched for developmental targets in that over one third of the corresponding genes encode TFs, morphogens or cytokines. In striking contrast, a second set of bivalent domains, occupied by PRC2 only, is actually under-represented for TF genes relative to the genome average, and shows weak conservation and retention of the PcG-associated chromatin marks. We suggest that the complete repertoire of PcG machinery is needed for full functionality of bivalent domains and associated chromatin in the epigenetic regulation of key developmental genes.

The data also suggest a potential model for understanding the initial recruitment of PcG complexes for the coordinated establishment of bivalent chromatin. In particular, we find that PRC2 association in ES cells is entirely restricted to sequences with high CpG content, the vast majority being annotated CpG islands. The status of a given CpG island – whether it carries PRC2 and bivalent H3K4me3/H3K27me3 chromatin or only H3K4me3 – correlates with underlying motif content. CpG islands with PRC2 show a striking depletion of transcriptional activator motifs and a modest enrichment of repressor motifs. Thus, PRC2 appears to localize to CpG islands that are transcriptionally silent in ES cells because they lack activating DNA sequence motifs.

CpG islands have been extensively correlated with trxG complexes and H3K4me3; recruitment of the former likely involves CXXC proteins with affinity for un-methylated CpG dinucleotides [15],[52],[53]. We propose that CpG islands by default similarly mediate PcG recruitment and catalysis of H3K27me3 in mammalian ES cells, except when the default is over-ridden by transcriptional activity. In this model, the extent of PcG/H3K27me3 and trxG/H3K4me3 at any given CpG island is determined by its baseline transcriptional status which is dictated by underlying motif content. The view that transcriptional status is upstream of PcG status in ES cells is consistent with the subtle transcriptional changes evident in PcG-deficient ES cells [9],[54]. Although our analyses do not shed light on the underlying mechanisms, PRC2 recruitment may also involve proteins with affinity for un-methylated CpGs or may be mediated indirectly through recognition of other histone modifications such as H3K4me3. In either case, active transcription within a locus would preclude stable PRC2 association and thereby restrict it to inactive CpG islands.

Large PRC2-positive CpG islands tend to also carry PRC1. The expansive regions of H3K27me3 associated with these islands may contribute to PRC1 recruitment via chromodomain proteins [2],[3]. As discussed above, bivalent domains that carry both PRC2 and PRC1 appear to have unique epigenetic regulatory properties. We therefore propose that large CpG islands depleted of activating motifs confer epigenetic regulation by recruiting both key PcG complexes in pluripotent cells. Such islands may thereby reflect mammalian memory elements analogous to Polycomb response elements in flies.

The tight correspondence between DNA sequence and PcG localization may have implications for important cellular processes, such as development and epigenetic reprogramming. Induced pluripotent stem (iPS) cells and ES cells exhibit nearly identical chromatin patterns, including the locations of bivalent domains [55],[56]. The sequences described above may function as templates for the robust assembly and appropriate positioning of PcG complexes and bivalent domains during pre-implantation development or the artificial reprogramming of somatic cells to iPS cells [1],[28].

What then might be the purpose of an initial chromatin state fully encoded by genetic sequence and an associated transcriptional program? Based on existing evidence, we suggest that PcG complexes and associated chromatin buffer the pluripotent ground state by reinforcing the repression of factors that induce differentiation. The initial chromatin architecture also appears poised for the dynamic expression changes that accompany differentiation and for the subsequent engagement of epigenetic controls to maintain lineage-specific transcriptional programs. Our analysis suggests that such epigenetic functions mainly apply to large bivalent CpG islands that also carry PRC1. It remains to be seen whether small PRC1-negative bivalent domains have distinct regulatory functions or are simply byproducts of the mechanisms that have evolved for establishment of the former.

Further studies are needed to determine the precise DNA elements and protein interactions that mediate PcG recruitment. As discussed above, the proposed central role for CG-rich sequences implies the involvement of CXXC domains or other proteins that recognize CG dinucleotides. However, several factors complicate the interpretation of our genomic findings. In particular, CpG islands are at least partly a consequence of reduced CpG deamination rates in regions that lack DNA methylation in the germ line [27]. PcG-occupied regions are largely un-methylated at the DNA level, at least in ES cells [57], and this could favor retention of CG-rich sequences. Thus, it remains possible that evolutionary dynamics and/or the generally high CpG content of target regions are masking other key sequence features.

Finally, it should be emphasized that our findings on the relationships among PRC2 and PRC1 and the sequences that underlie their genomic localizations pertain specifically to ES cells. PcG complexes show remarkable tissue-specificities in terms of their expression levels, stoichiometry and localization [2],[3],[11],[12]. Further study is needed to understand how the genomic localizations and regulatory functions of PcG complexes vary with differentiation, lineage specification, environment, and disease.

## Materials and Methods

### Cell Culture

Mouse v6.5 (genotype 129SvJae×C57BL6, male, passages 10–15) ES cells were cultured on fibroblast feeders in DMEM (Sigma) with 15% fetal bovine serum (Hyclone), GlutaMax (Invitrogen), MEM non-essential amino acids (Invitrogen), pen/strep (Invitrogen), ESGRO (Chemicon) and 2-mercaptoethanol (Sigma), incubating at 37°C, 5% CO_2_ [16]. Prior to harvest, these cells were passaged 2–3 times on feeder-free gelatinized tissue culture plates. A transgenic ES cell line expressing a fusion between Ring1B and biotin ligase recognition peptide from the endogenous Ring1B locus and the BirA biotin ligase from the Rosa26 locus (H.K., unpublished) was cultured as described above.

Human H9 (female, passage 45) ES cells were cultured as described [58] and at http:/www.WiCell.org. Briefly, the human ES cells were cultivated on irradiated MEFs (strain DR4) in Knockout DMEM (Invitrogen) containing 10% Knockout Serum Replacement (Invitrogen), 10% Plasmanate (Bayer Healthcare), GlutaMax (2 mM), pen/strep, MEM non-essential amino acids (0.1 mM), 10 ng/ml β-FGF (Invitrogen) and 2-mercaptoethanol. Cells were incubated at 37°C, 5% CO_2_. MEF-free ES cells were used for analysis. MEF-free culture was prepared in the following manner: First, MEFs were depleted at the time of trypsin passaging through brief transfer (thirty minutes) of hES cells onto gelatin-coated plates. MEF-subtracted ES cells were then propagated on plates coated with Matrigel (Invitrogen). ES cells grown on Matrigel were supported with the aforementioned human ES cell medium that had first been conditioned on MEFs for 24 hours. Fresh β-FGF was added to the conditioned medium immediately prior to use.

### Generation of Flag-Bmi1 mES Cells

Doxycyclin-inducible Flag-Bmi1 transgenic ES cell line was generated by PCR amplifying a 1× flag tagged Bmi1 ORF (Addgene) with primers that incorporate a 3× flag tag as well as EcoRI and XbaI restriction enzyme sites (5′-GGAATTCCACCATGGACTACAAAGACCATGACGGTGATTATAAAGATCATGATATCGACTACAAGGACG-3′, 5′- GCTCTAGAGCACCAGATGAAGTTGCTGATGACCCATTTAGTGATGATTTT-3′). This was cloned into the pLox vector (pPGK-loxP-neoEGFP) and incorporated into Ainv15 mouse ES cells using a cre recombinase expression vector as previously described [59]. Flag-Bmi1 ES cells were cultured similarly to wild-type mES cells as described above. Prior to harvest, Flag-Bmi1 expression was induced by incubating with 1 µg/ml of Doxycycline for two days on gelatinized culture plates.

### Chromatin Immunoprecipitation and Antibodies

ChIP experiments for H3K4me3, H3K27me3 and H3K36me3, Ring1B and Flag-Bmi1 were carried out as described [15],[16]. ES cells were crosslinked in 1% formaldehyde, lysed and sonicated with either a Branson 250 Sonifier (mouse ES cells) or a Diagenode bioruptor (human ES cells) to obtain chromatin fragments in a size range between 200 and 700 bp. Solubilized chromatin (whole cell lysate or ‘WCE’) was diluted in ChIP dilution buffer (1∶10) and incubated with antibody overnight at 4°C. Protein A sepharose beads (Sigma) were used to capture the antibody-chromatin complex and washed with low salt, LiCl, as well as TE (pH 8.0) wash buffers. Enriched chromatin fragments were eluted at 65°C for 10 min, subjected to crosslink reversal at 65°C for 5 hrs, and treated with Proteinase K (1 mg/ml), before being extracted by phenol-chloroform-isoamyl alcohol, and ethanol precipitated. ChIP DNA was then quantified by Quant-iT Picogreen dsDNA Assay kit (Invitrogen).

ChIP experiments for Ezh2 and Suz12 were carried out on nuclear preps. Crosslinked ES cells were incubated in swelling buffer (0.1 M Tris pH 7.6, 10 mM KOAc, 15 mM MgOAc, 1% NP40), on ice for twenty minutes, passed through a 16G needle 20 times and centrifuged to collect nuclei [60]. Isolated nuclei were then lysed, sonicated and immunoprecipitated as described above.

BioChIP assays were carried out using transgenic Ring1B-Biotin ligase recognition peptide ES cells (above). Nuclei were isolated, lysed and sonicated as described above. Dynabeads M-280 Streptavidin (Invitrogen 112.05D) were used to capture biotinylated Ring1B-DNA complex. Beads were washed with a 2% SDS buffer and a high salt buffer (50 mM HEPES, pH 7.5, 1 mM EDTA, 500 mM NaCl, 1% Triton X-100, 0.1% Deoxycholate), in addition to the regular washes. Elution and cross-link reversal were done simultaneously by incubating Dynabeads in 300 mM NaCl at 65°C overnight [46]. DNA was isolated as described above.

Antibodies used in this study include anti-H3K4me3 (Abcam ab8580), anti-H3K27me3 (Upstate 07-449), anti-H3K36me3 (Abcam ab9050), anti-Ezh2 (Active Motif 39103), anti-Suz12 (Abcam ab12073), anti-Ring1B [61] and anti-Flag (M2) (Sigma F1804). Details on antibody specificity are provided in Text S1.

### Sequencing Library Preparation and Illumina/Solexa Sequencing

Library preparation and ultra high-throughput sequencing were carried out as described [16]. Briefly, one to ten nanograms (ng) of ChIP DNA were end-repaired and 5′phosphorylated using END-It DNA End-Repair Kit (Epicentre). We then followed steps four through seven of Illumina standard sample prep protocol (v1.8) using Genomic DNA Sample Prep Kit (Illumina) with minor modifications. A single Adenine was added to 3′ ends by Klenow (3′→5′ exo^−^), and double-stranded Illumina Adapters were ligated to the ends of the ChIP fragments. Adapter-ligated ChIP DNA fragments between 275 bp to 700 bp were gel-purified and subjected to 18 cycles of PCR. Prepared libraries were quantified using PicoGreen and sequenced on the Illumina Genome Analyzer per standard operating procedures.

### Read Alignment and Generation of Density Maps and Modified Intervals

Sequence reads (36 bases) from each ChIP experiment were compiled, post-processed and aligned to the appropriate reference genome using a general purpose computational pipeline as described previously [16]. Aligned reads are used to estimate the number of end-sequenced ChIP fragments that overlap any given genomic position (at 25-bp resolution). For each position, we counted the number of reads that are oriented towards it and closer than the average length of a library fragment (∼300 bp). The result is a high-resolution density map that can be viewed through the UCSC Genome Browser [62] and is used for downstream analyses. Prior comparisons to microarray analysis and quantitative real-time PCR have shown that ChIP-Seq density maps accurately reflect enrichment [16]. ChIP-Seq data can be accessed at http://www.broad.mit.edu/seq_platform/chip/.

We used a Hidden Markov Model (HMM) to demarcate chromosomal segments likely to be enriched for a given chromatin modification or PcG protein [16]. In order to model ChIP-Seq read density variations along the genome, we define four observed states: masked, low density, medium density, and high density. This discretization of the data into the four states was based on the signal intensity in known modified regions versus known unmodified regions as determined in prior ChIP-Seq, microarray and ChIP-PCR analyses [15],[16], and adjusted for each sample. The model was then used to discriminate enriched and unenriched intervals genome wide. In order to more properly classify enriched regions containing several short interspersed peaks and facilitate subsequent analyses intervals within 2 kb were merged.

### Promoter Classification and Definition of Gene and Transcript Intervals

We defined 17760 mouse and 18522 human promoters for 17442 and 17383 genes, respectively, as the sequences between −0.5 kb and +2.0 kb of the annotated transcription start site, using the mouse mm8 and human hg18 genome builds. Transcripts were defined for these genes as the range from transcription start to end [62]. To identify regions enriched for histone marks or chromatin-associated proteins, we generated a null-hypothesis background model by dividing the alignable parts of each chromosome into 200 bp bins and randomly redistributing the reads aligned on this chromosome. Based on a histogram of the cumulative distribution of reads per bin, a cutoff threshold was determined. Stability of the calculated background cutoff threshold was confirmed through 1000 independent simulations for each ChIP-Seq track and showed remarkable invariance. For promoters, a 200 bp sliding window was moved across the 2.5 kb promoter region and the ratio of median read density over background was calculated. The maximum enrichment achieved in any window at this promoter site was then used for further analysis. Maximum enrichment cutoff thresholds were determined empirically for all tracks, and promoters were then classified based on the maximum enrichment for the various histone marks and PcG proteins. The same procedure was applied to a pan-H3 (modification-insensitive) ChIP-Seq dataset as control where virtually no significant enrichment over background was found. Ring1B-positive bivalent promoters were defined based on normalized ChIP-Seq signal and comprise 40% of all bivalent promoters. A set of Ring1B-negative bivalent promoters was also defined based on absence of ChIP-Seq enrichment, and includes another 40% of all bivalent promoters. The remaining bivalent promoters (20%) with indeterminate Ring1B ChIP-Seq signals were excluded from this analysis.

For conservation analyses of human and mouse promoter states, we used NCBI HomoloGene (build 58) gene clusters to assign orthologous human promoters and transcripts to the 17442 mouse promoters and transcripts, yielding a set of 13200 orthologous promoters and 13625 orthologous transcripts for which human and mouse chromatin state could be compared (ftp://ftp.ncbi.nih.gov/pub/HomoloGene/). Genes with multiple start sites were excluded from this analysis. Promoters were associated with CpG states as described previously [16].

For comparison of Ezh2 and Ring1B occupancy at target genes, a reduced Ezh2 read set was generated by randomly selecting the same number of reads that were available for Ring1B from the full Ezh2 read pool (∼3.5 million). Read mapping to the mouse genome and analysis of promoter state were performed as described above.

### Real-Time PCR

PCR primer pairs were designed to amplify designated genomic regions using Primer3 (http://fokker.wi.mit.edu/primer3/input.htm). Real-time PCR assays were carried out on ABI 7000 or 7500 detection systems. We used Quantitect SYBR green PCR mix (Qiagen) with 0.1 ng ChIP or 0.1 ng un-enriched input DNA (WCE) as template. Log_2_ enrichment was calculated from geometric means obtained from three independent ChIP experiments, each evaluated by duplicate PCR assays. Background was subtracted by normalizing over negative genomic control.

### Gene Expression Analysis

Gene expression data for Ring1A/B-dKO (*Ring1A* ^−/−^;*Ring1B* ^fl/fl^;*Rosa26*::*CreERT2*) ES cells (2 day post-tamoxifen treatment and no-treatment control, H. Koseki unpublished data) and Eed KO ES cells (Eed −/− and control Eed+/+ ES) [13], acquired with Affymetrix Mouse Genome 430 2.0 Arrays, were normalized using the Genepattern expression data analysis package (http://www.broad.mit.edu/cancer/software/genepattern). CEL files were processed with RMA, quantile normalization and background correction [63]. For a given comparison (Ring1A/B-dKO vs control; or Eed −/− vs +/+), we only considered probes in which at least one of the experiments had a “P” significance call. Fold changes were calculated for each passing probe. Genes with multiple corresponding probes were assigned the geometric average fold change value. Gene expression data for mouse v6.5 mES and NPCs were obtained from previously published Affymetrix mRNA profiles [16].

### Gene Class Enrichment Analysis

Gene ontology (GO) functional annotation for the Ring1B positive and negative sets was done using DAVID analysis tool (http://david.abcc.ncifcrf.gov/home.jsp). P-values were adjusted for multiple hypothesis testing using Bonferroni correction.

### CG Content and Motif Enrichment Analysis

The HMM described above was used to define enriched intervals for each modification or chromatin protein from the mouse ES cell ChIP-Seq data. We determined the extent to which Ezh2 intervals (and those for other epitopes) overlap with CG-rich sequences. CpG island coordinates were obtained from the UCSC Genome Browser [62]. We identified all Ezh2 intervals that overlap these CpG island coordinates within 500 bp. Next, the EMBOSS analysis package [64] was used to determine the portion of remaining Ezh2 intervals overlapping a ‘mini’ CpG island defined as a 100 bp window with at least 50% GC content and an O∶E ratio >0.6 (instead of the standard CpG island window of 200 bp).

We next classified CpG islands according to their chromatin state (e.g., Ezh2-positive v. Ezh2-negative, H3K4me3 v. bivalent). This was done by computing the median ChIP-Seq read density across each defined CpG island, and setting thresholds using a null background model of randomized reads. For these analyses we excluded CpG islands that fall within unalignable regions, typically due to low complexity sequence, and thus could not be evaluated by ChIP-Seq (<7% of all CpG islands). To maximize discriminatory power, we excluded intermediate CpG islands with sub-threshold Ezh2 signal.

We computed median values and distributions for length, CG density and observed-to-expected ratio for the different CpG island sets, and also evaluated nucleotide content by calculating the frequencies of all 16 dinucleotide combinations. Conservation scores were determined for each CpG island by aligning the regions between mouse and rat, and performing a dinucleotides level comparison of the conservation between the two species. Both CpG and non-CpG dinucleotides were conserved at slightly higher levels in the Ezh2-bound CpG islands (Figure S7).

We next screened the CpG island sets for TF motif occurrences. 668 position weight matrices (PWMs) were obtained from the Jaspar (Release 3.0 [34]) and TRANSFAC (Release 9.4; [35]) databases, excluding any non-vertebrate factors. We prepared sets of Ezh2-positive and Ezh2-negative sequences by extracting each CpG island along with flanking sequence equal to 50% of its length. The MAST algorithm [36] was then used to search for significant PWM matches (p<5e-5) in the Ezh2-positive and negative sets. Occurrences were length-normalized and used to calculate ratios that reflect the enrichment in the Ezh2-positive set relative to the Ezh2-negative set, or vice versa. We identified significantly over-represented motifs using Fisher's exact test with Bonferroni-adjusted p-values. These candidate motifs were then scrambled, re-scored, and excluded if any enrichment was observed in the scramble.

We used a clustering algorithm to collapse similar motifs identified as enriched in one of the sets to a single consensus sequence [65]. This was necessary due to high motif redundancy in the databases. After clustering, all intra-cluster motif occurrences overlapping by more than 50% were counted as a single instance. Expression values for corresponding DNA binding proteins were determined from previously published Affymetrix mRNA profiles for v6.5 ES cells [16].

A simple count-based model was used to determine the extent to which motif occurrences are predictive of Ezh2 status. The motif content which allowed for maximum discrimination in mouse is as follows: a CpG island was predicted to be Ezh2-positive if it either (i) contained >8 ‘Ezh2-positive’ motifs or (ii) contained >4 ‘Ezh2-positive’ motifs and <2 ‘Ezh2-negative’ motifs. Ezh2 status in human was predicted using the motifs identified in mouse but with the following metric: a CpG island was predicted to be Ezh2-positive if it contained >15 ‘Ezh2-positive’ motifs and <2 ‘Ezh2-negative’ motifs.

In order to quantify Ring1B presence in CpG islands, we considered the distribution of ChIP-Seq reads in control regions. We specifically used all alignable, H3K4me3-only CpG islands as our null hypothesis background model. The distribution of Ring1B ChIP-Seq read densities across these islands was calculated and a threshold was set to minimize the false positive detection rate. We then calculated Ring1B ChIP-Seq read density in sliding 200 bp windows in all Ezh2-positive CpG islands, with a CpG island assigned the maximum enrichment in any of its 200 bp windows. For maximum discriminatory power, we excluded 20% of CpG islands with sub-threshold Ring1B signal. Ring1B status was predicted using the length of CpG-richness in PRC2-positive CpG islands. Islands were predicted to be Ring1B-positive if they were either >1200 bp or within 2 kb of another CpG island.

## Supporting Information

Figure S1 Comparison of chromatin states in mouse and human ES cells. (A) Conservation of H3K4me3 for 13,200 transcription start sites between human and mouse. Dashed lines indicate cutoff thresholds used to binarize the data for further analysis. Genes that carry H3K4me3 are likely to be conserved (upper right quadrant), as are those that are not marked (lower left quadrant). Less than 12% of genes are differentially methylated between human and mouse (upper left and lower right quadrants). (B) Conservation of H3K27me3 for the same regions used in (A). Most genes in both mouse and human are not marked with H3K27me3 (bottom left quadrant). Only slightly more than half the genes that carry H3K27me3 in mouse do so in human also. (upper and lower right quadrant). (C) H3K4me3 vs. H3K27me3 plotted for 17,760 mouse genes reveal three prominent marks in ESC: H3K4me3 only, (lower right quadrant), H3K4me3+H3K27me3/bivalent (upper right quadrant) and “no mark” (lower left quadrant). Very few genes are marked with H3K27me3 only (upper left quadrant).
(3.85 MB PDF)

Figure S2 Quantitative PCR enrichment for Ezh2 ChIP, Ring1B bioChIP and Flag-Bmi1 ChIP. (A) Plot shows Log2 ChIP-qPCR enrichment of Ezh2 in mouse v6.5 ES cells at bivalent gene promoters. Included are promoters classified as PRC2-bound (orange) or PRC2-unbound (yellow) by ChIP-Seq. (B) Plot shows Log2 enrichment of Ring1B bioChIP-qPCR in transgenic mouse ES cells expressing biotin-tagged Ring1B (mES*) at bivalent promoters classified by ChIP-Seq as PRC1-bound (purple) or PRC1-unbound (blue). H3K4me3 only genes are green. (C) Plot shows fold enrichment of Flag ChIP-qPCR in transgenic mouse ES cells expressing Flag-tagged Bmi1 (mEŜ) at bivalent promoters classified by ChIP-Seq as PRC1-bound (purple) or PRC1-unbound (blue).
(0.31 MB PDF)

Figure S3 Chromatin states of species-specific factors from ES cell Pathways. Divergent chromatin states of species-specific factors in transcription and signaling pathways observed in mouse and human ES cells reflect known distinctive biological functions between the two pluripotency models.
(0.28 MB PDF)

Figure S4 Expression analysis in PRC2 wild-type (WT) and knock-out (KO) mouse ES cells. Expression changes for all genes, Ring1B-positive bivalent and Ring1B-negative bivalent genes in PRC2 knock-out (Eed−/−) mouse ES cells.
(0.15 MB PDF)

Figure S5 Analysis of the CG-richness of HMM-defined intervals of H3K4me3, H3K27me3, H3K36me3, H3K9me3, H3K20me3, and Ezh2. (A) The fraction of intervals that either directly overlap or are within 500 bp of a CpG island. (B) The maximum CpG observed-to-expected ratio in any 200 bp window within the interval. The dashed line marks 0.6, one of the criteria used to define a CpG island.
(0.21 MB PDF)

Figure S6 Comparison of Ezh2-positive and Ezh2-negative CpG islands. No marked difference was observed in CpG observed-to-expected ratio (A), percent CpG (B), or percent GC (C), whereas Ezh2-positive CpG islands tend to be longer (median 721 bp vs 526 bp; D).
(0.22 MB PDF)

Figure S7 Conservation of Ezh2-bound and Ezh2-unbound dinucleotides between rat and mouse. Aligning regions in rat (rn4) for both classes of CpG island were identified, and a dinucleotide level comparison was performed on the conservation between the two species. Both non-CpG (A) and CpG (B) dinucleotides were conserved at slightly higher levels in the Ezh2-bound CpG islands than in those islands that did not bind Ezh2.
(0.70 MB PDF)

Figure S8 Motif clusters and their respective enrichment p-values for Ezh2-positive and Ezh2-negative CpG islands. The top ranking motifs (and their Bonferroni-corrected p-values from Fisher's exact test) for Ezh2-negative (A) and positive (B) CpG islands. The motifs were clustered and collapsed to reduce redundancy.
(0.49 MB PDF)

Figure S9 Length of CpG islands in Ring1B-positive and Ring1B-negative bivalent promoters. Ring1B-positive bivalent CpG islands are larger than bivalent CpG islands that are only bound by PRC2.
(0.12 MB PDF)

Table S1 List of ChIP-Seq datasets showing numbers of aligned reads.
(0.28 MB PDF)

Table S2 Chromatin states of analyzed promoters in mES cells.
(3.72 MB XLS)

Table S3 Chromatin states of analyzed promoters in hES cells (Microsoft Excel file).
(2.81 MB XLS)

Table S4 Comparison of chromatin states of analyzed promoters between mES and hES cells.
(1.69 MB XLS)

Table S5 PCR primers used for Ezh2, Ring1B and Flag-Bmi1 ChIP-qPCR in mouse ES cells.
(0.61 MB PDF)

Text S1 Supporting information on the specificity of antibodies. Western blots using mouse ES cell protein extracts demonstrate the specificity of anti-Ring1B and anti-Ezh2 (Active Motif 39103), antibodies used in this study. *Indicates the expected molecular weight. Previous publications that demonstrate the specificity of the antibodies used are listed.
(1.83 MB PDF)

Text S2 Relevant references for transcription factors (TFs) that correspond to implicated motifs and are active in ES cells.
(0.63 MB PDF)
