# Supplementary figures and images for: Genomewide Analysis of PRC1 and PRC2 Occupancy Identifies Two Classes of Bivalent Domains
Source: PLoS Genet. 2008 Oct 31;4(10):e1000242. doi: 10.1371/journal.pgen.1000242 (PMC2567431; doi:10.1371/journal.pgen.1000242)

Figure S1. Comparison of chromatin states in mouse and human ES cells.

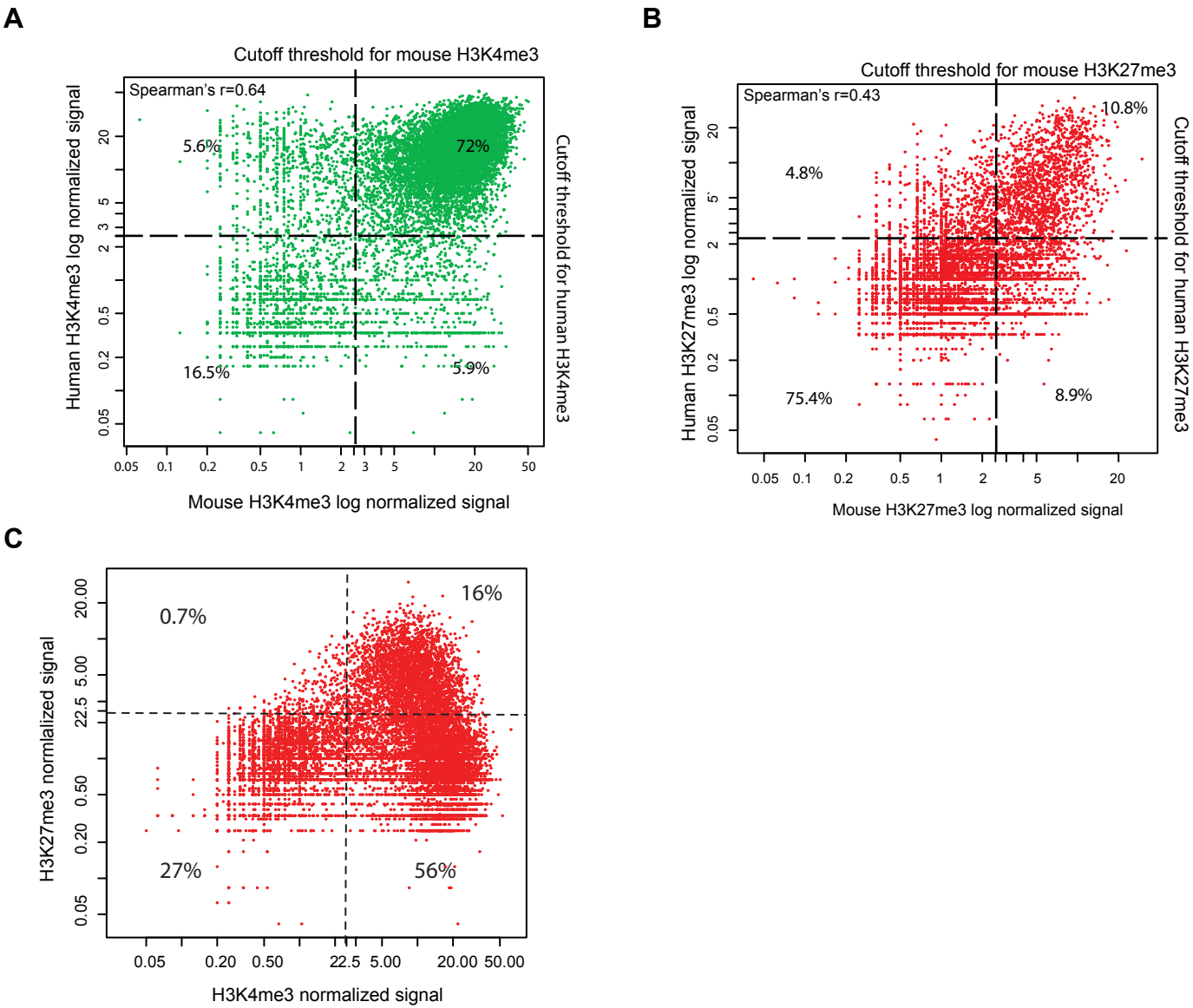

Supplement: Figure S1 — Comparison of chromatin states in mouse and human ES cells. (A) Conservation of H3K4me3 for 13,200 transcription start sites between human and mouse. Dashed lines indicate cutoff thresholds used to binarize the data for further analysis. Genes that carry H3K4me3 are likely to be conserved (upper right quadrant), as are those that are not marked (lower left quadrant). Less than 12% of genes are differentially methylated between human and mouse (upper left and lower right quadrants). (B) Conservation of H3K27me3 for the same regions used in (A). Most genes in both mouse and human are not marked with H3K27me3 (bottom left quadrant). Only slightly more than half the genes that carry H3K27me3 in mouse do so in human also. (upper and lower right quadrant). (C) H3K4me3 vs. H3K27me3 plotted for 17,760 mouse genes reveal three prominent marks in ESC: H3K4me3 only, (lower right quadrant), H3K4me3+H3K27me3/bivalent (upper right quadrant) and “no mark” (lower left quadrant). Very few genes are marked with H3K27me3 only (upper left quadrant). (3.85 MB PDF) [file pgen.1000242.s001.pdf]

Figure S2 Quantitative PCR enrichment for Ezh2 ChIP, Ring1B bioChIP and Flag-Bmi1 ChIP.

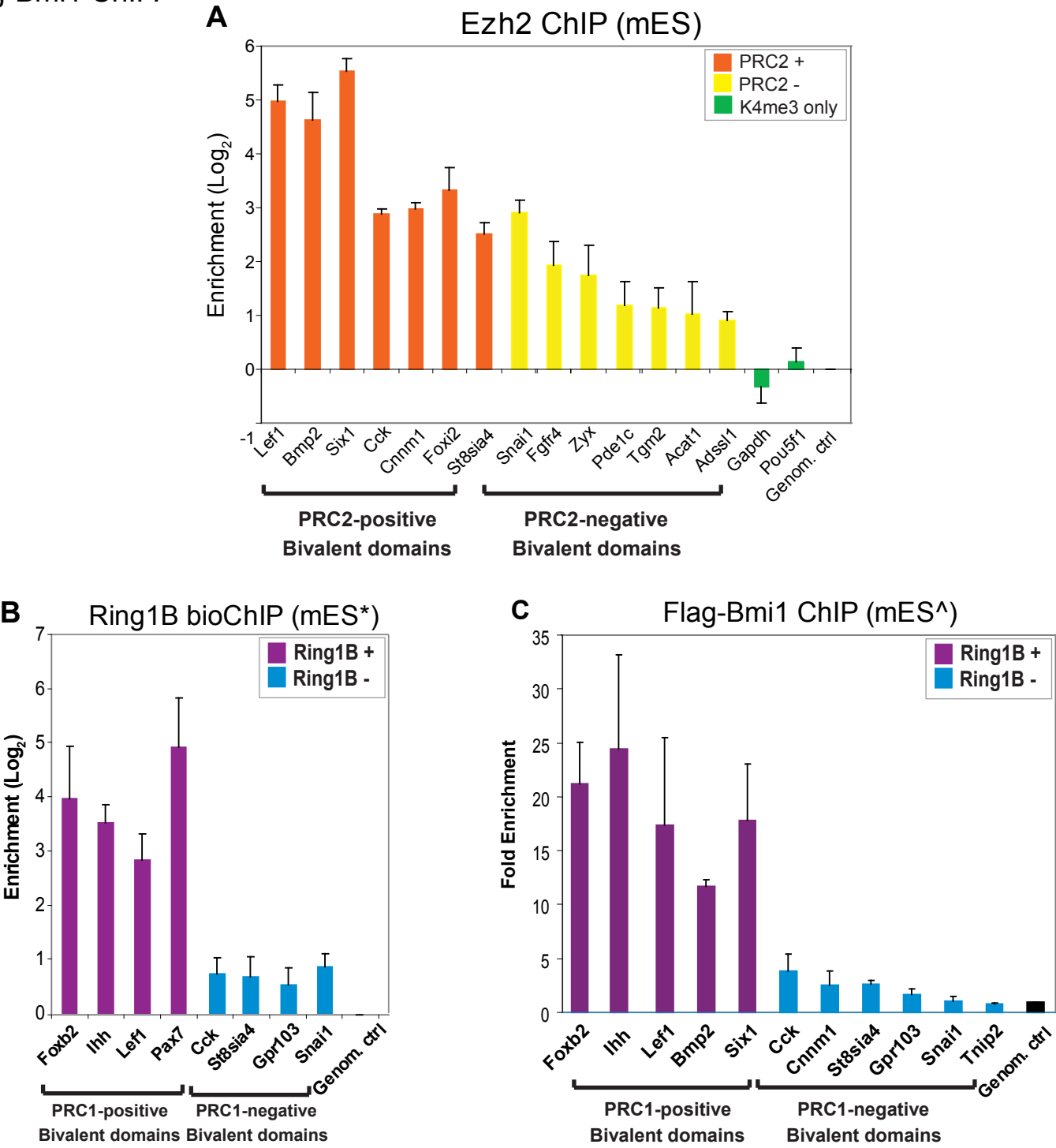

Supplement: Figure S2 — Quantitative PCR enrichment for Ezh2 ChIP, Ring1B bioChIP and Flag-Bmi1 ChIP. (A) Plot shows Log2 ChIP-qPCR enrichment of Ezh2 in mouse v6.5 ES cells at bivalent gene promoters. Included are promoters classified as PRC2-bound (orange) or PRC2-unbound (yellow) by ChIP-Seq. (B) Plot shows Log2 enrichment of Ring1B bioChIP-qPCR in transgenic mouse ES cells expressing biotin-tagged Ring1B (mES*) at bivalent promoters classified by ChIP-Seq as PRC1-bound (purple) or PRC1-unbound (blue). H3K4me3 only genes are green. (C) Plot shows fold enrichment of Flag ChIP-qPCR in transgenic mouse ES cells expressing Flag-tagged Bmi1 (mEŜ) at bivalent promoters classified by ChIP-Seq as PRC1-bound (purple) or PRC1-unbound (blue). (0.31 MB PDF) [file pgen.1000242.s002.pdf]

Figure S4. Expression analysis in PRC2 wild-type (WT) and knock-out (KO) mouse ES cells.

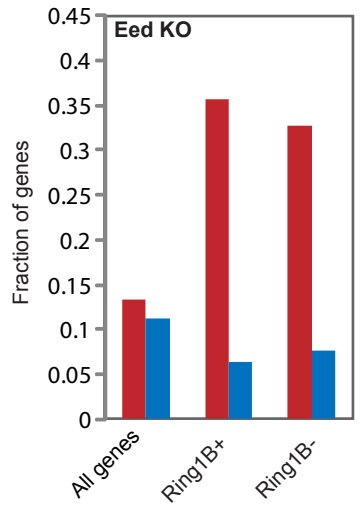

Supplement: Figure S4 — Expression analysis in PRC2 wild-type (WT) and knock-out (KO) mouse ES cells. Expression changes for all genes, Ring1B-positive bivalent and Ring1B-negative bivalent genes in PRC2 knock-out (Eed−/−) mouse ES cells. (0.15 MB PDF) [file pgen.1000242.s004.pdf]

Figure S6. Comparison of Ezh2-positive and Ezh2-negative CpG islands.

A

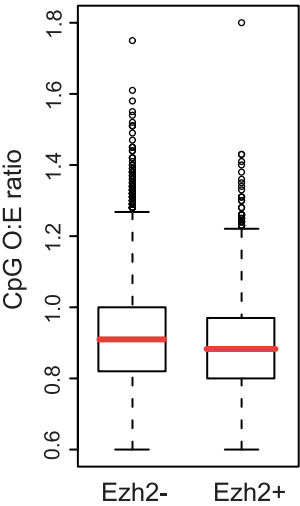

B

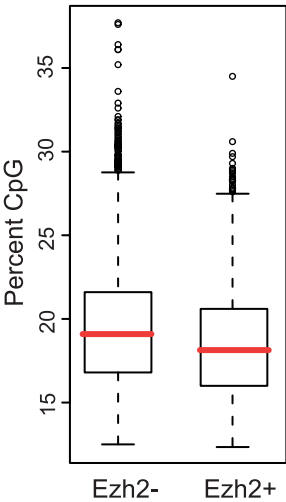

C

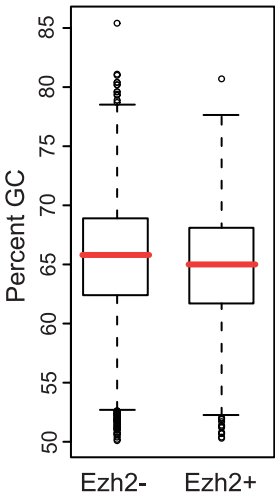

D

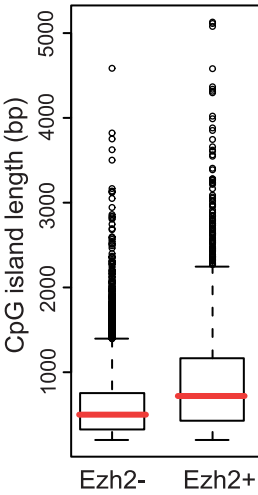

Supplement: Figure S6 — Comparison of Ezh2-positive and Ezh2-negative CpG islands. No marked difference was observed in CpG observed-to-expected ratio (A), percent CpG (B), or percent GC (C), whereas Ezh2-positive CpG islands tend to be longer (median 721 bp vs 526 bp; D). (0.22 MB PDF) [file pgen.1000242.s006.pdf]

Figure S7. Conservation of Ezh2-bound and Ezh2-unbound dinucleotides between rat and mouse.

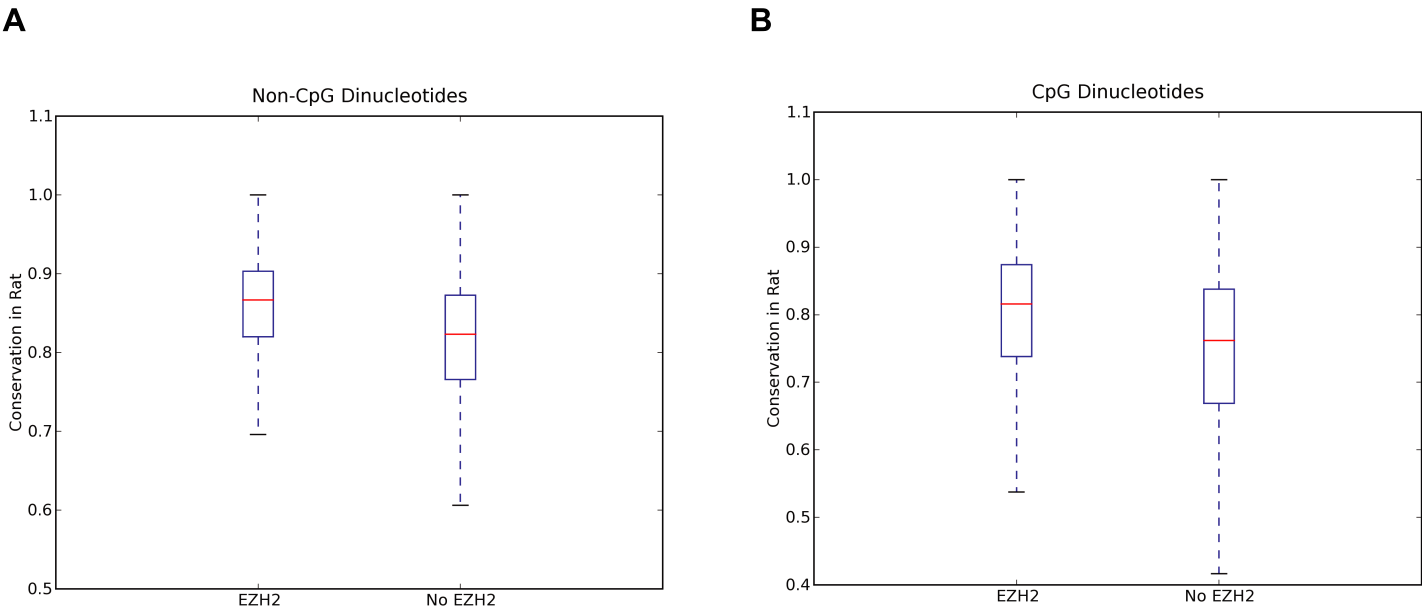

Supplement: Figure S7 — Conservation of Ezh2-bound and Ezh2-unbound dinucleotides between rat and mouse. Aligning regions in rat (rn4) for both classes of CpG island were identified, and a dinucleotide level comparison was performed on the conservation between the two species. Both non-CpG (A) and CpG (B) dinucleotides were conserved at slightly higher levels in the Ezh2-bound CpG islands than in those islands that did not bind Ezh2. (0.70 MB PDF) [file pgen.1000242.s007.pdf]

Figure S9. Length of CpG islands in Ring1B-positive and Ring1B-negative bivalent promoters.

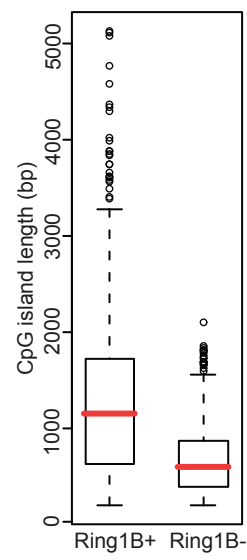

Supplement: Figure S9 — Length of CpG islands in Ring1B-positive and Ring1B-negative bivalent promoters. Ring1B-positive bivalent CpG islands are larger than bivalent CpG islands that are only bound by PRC2. (0.12 MB PDF) [file pgen.1000242.s009.pdf]
