# Supplementary material for: Genomewide Analysis of PRC1 and PRC2 Occupancy Identifies Two Classes of Bivalent Domains
Source: PLoS Genet. 2008 Oct 31;4(10):e1000242. doi: 10.1371/journal.pgen.1000242 (PMC2567431; doi:10.1371/journal.pgen.1000242)
Supplement: Figure S3 — Chromatin states of species-specific factors from ES cell Pathways. Divergent chromatin states of species-specific factors in transcription and signaling pathways observed in mouse and human ES cells reflect known distinctive biological functions between the two pluripotency models. (0.28 MB PDF) [file pgen.1000242.s003.pdf]

Figure S3. Chromatin states of species-specific factors from ES cell Pathways.

Chromatin States of species-specific factors from ES Cell Pathways		
	Mouse ES Cells (v6.5)	Human ES Cells (H9)
FGF Signaling		
FGF2	Bivalent	K4
FGF8	Bivalent	Bivalent
FGF12	Bivalent	Bivalent
FGFR2	bivalent	K4
FGFR3	bivalent	K4
FGFR4	bivalent	K4
Spry	K4	K4
Nodal/Activin		
Nodal	K4	K4
Lefty2	Bivalent	K4
Lefty1	Bivalent	K4
Inhba	Bivalent/Bivalent	Bivalent/K4
Acvr2b	K4	K4
FSTL1	K4	K4
Lif/Stat Pathway		
LifR	K4	K4
Stat3	K4	K4
Socs-1	Bivalent	K4
ICM Specific		
Gbx2	K4	Bivalent
FGF4	K4	Bivalent
