# Supplementary material for: Genomewide Analysis of PRC1 and PRC2 Occupancy Identifies Two Classes of Bivalent Domains
Source: PLoS Genet. 2008 Oct 31;4(10):e1000242. doi: 10.1371/journal.pgen.1000242 (PMC2567431; doi:10.1371/journal.pgen.1000242)

Figure S5. Analysis of the CG-richness of HMM-defined intervals of H3K4me3, H3K27me3, H3K36me3, H3K9me3, H3K20me3, and Ezh2.

A

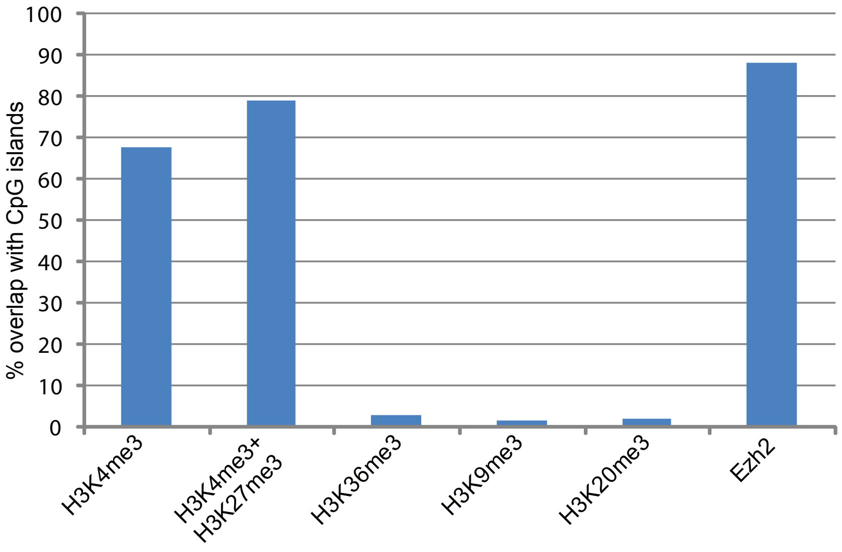

B

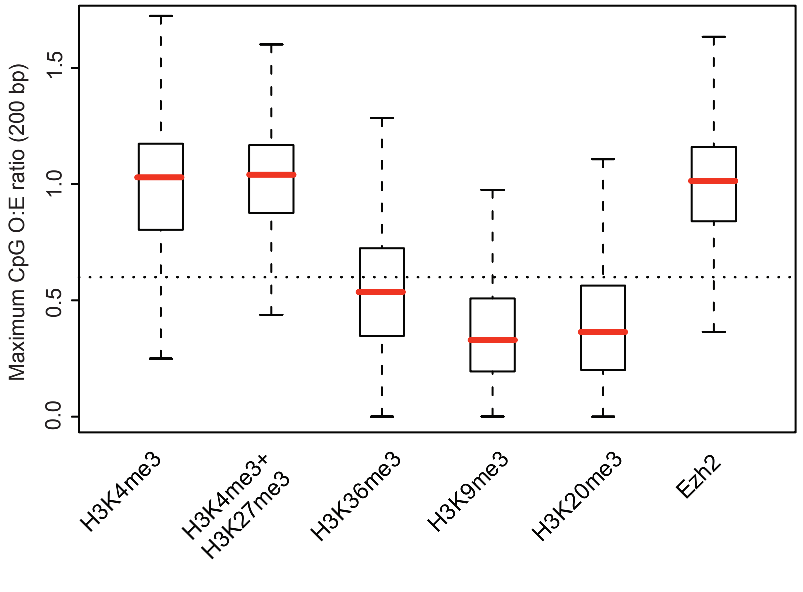

Supplement: Figure S5 — Analysis of the CG-richness of HMM-defined intervals of H3K4me3, H3K27me3, H3K36me3, H3K9me3, H3K20me3, and Ezh2. (A) The fraction of intervals that either directly overlap or are within 500 bp of a CpG island. (B) The maximum CpG observed-to-expected ratio in any 200 bp window within the interval. The dashed line marks 0.6, one of the criteria used to define a CpG island. (0.21 MB PDF) [file pgen.1000242.s005.pdf]
