# Supplementary material for: Genomewide Analysis of PRC1 and PRC2 Occupancy Identifies Two Classes of Bivalent Domains
Source: PLoS Genet. 2008 Oct 31;4(10):e1000242. doi: 10.1371/journal.pgen.1000242 (PMC2567431; doi:10.1371/journal.pgen.1000242)

Figure S8. Motif clusters and their respective enrichment p-values for Ezh2-positive and Ezh2-negative CpG islands.

A

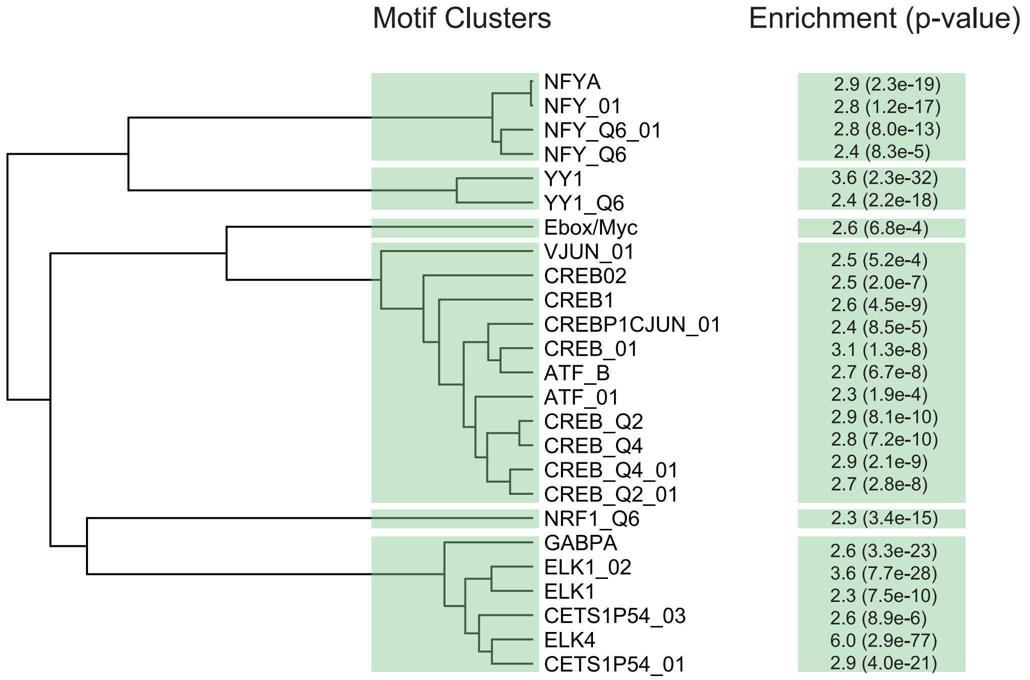

B

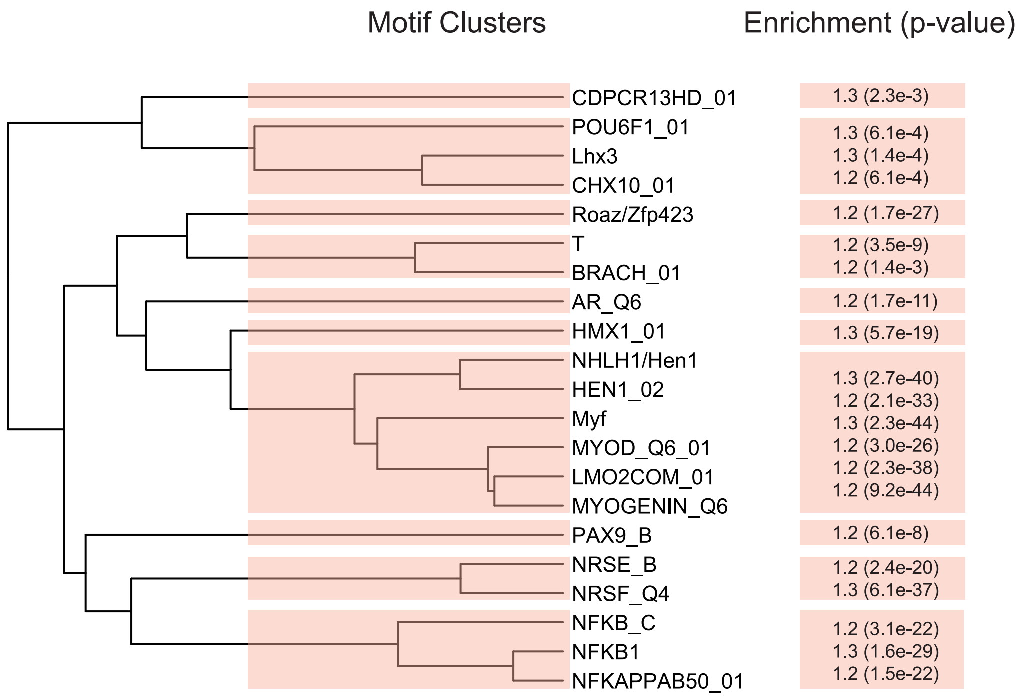

Supplement: Figure S8 — Motif clusters and their respective enrichment p-values for Ezh2-positive and Ezh2-negative CpG islands. The top ranking motifs (and their Bonferroni-corrected p-values from Fisher's exact test) for Ezh2-negative (A) and positive (B) CpG islands. The motifs were clustered and collapsed to reduce redundancy. (0.49 MB PDF) [file pgen.1000242.s008.pdf]
