# Supplementary material for: Genomewide Analysis of PRC1 and PRC2 Occupancy Identifies Two Classes of Bivalent Domains
Source: PLoS Genet. 2008 Oct 31;4(10):e1000242. doi: 10.1371/journal.pgen.1000242 (PMC2567431; doi:10.1371/journal.pgen.1000242)
Supplement: Table S1 — List of ChIP-Seq datasets showing numbers of aligned reads. (0.28 MB PDF) [file pgen.1000242.s010.pdf]

Table S1. List of ChIP-Seq datasets showing numbers of aligned reads.

Cell Type[1]	Epitope	# Aligned Reads
mES cells	Ezh2	7006533
	Suz12	8413470
	Ring1B	3482313
hES cells	H3K4me3	7644200
	H3K27me3	6572966
	H3K36me3	7630514
	EZH2	11114357
	RING1B	1607409

[1] **mES cells are from genotype 129SVJae x C57BL/6 F1 mice; hES cells are H9.**
