# Supplementary material for: Genomewide Analysis of PRC1 and PRC2 Occupancy Identifies Two Classes of Bivalent Domains
Source: PLoS Genet. 2008 Oct 31;4(10):e1000242. doi: 10.1371/journal.pgen.1000242 (PMC2567431; doi:10.1371/journal.pgen.1000242)
Supplement: Table S5 — PCR primers used for Ezh2, Ring1B and Flag-Bmi1 ChIP-qPCR in mouse ES cells. (0.61 MB PDF) [file pgen.1000242.s014.pdf]

Table S5. PCR primers used for Ezh2, Ring1B and Flag-Bmi1 ChIP-qPCR in mouse ES cells.

Gene Name	Forward	Reverse
**Acat1**	CTCCTGGGCCTACAGAGAGA	CGTGTGTCAGTCCACCTGTC
**Adssl1**	CCTCCAGATTAACGGCAAAG	AGGACTAGGGTTTGGGGTTG
**Bmp2**	CCGATCACCTCTCTTCCTCA	CTGGGCTTCTGTTGCTTTTC
**Cck**	AGACATACGCCGCTCTTCAT	ACTTCTGTGTGCGGGACTTT
**Cnnm1**	TGCTTGACTTCGCCACTGTA	AAAGGCCAAGTCTTTGACGA
**Fgfr4**	GGGTGGGGGCATTAAAGTAA	GTTGTTCACCCTCCTGCACT
**Foxb2**	AGTGGCACAGAGCCTTGACT	CATGTTGGCGTGATGGATT
**Foxi2**	CTTCTACAAGCGCAGCAAGG	TGTGCTGCTGTTTACCTGGA
**Gapdh**	AGCATCCCTAGACCCGTACAGT	GGGTTCCTATAAATACGGACTGC
**Gpr103**	GCAGATCACTGAGTGCCAGA	CTGGCTGGAGCACTCATTTT
**Ihh**	GCTGGGCTGTGAGAAATGTT	CTGGCAGCTCAGGTCAAATC
**Lef1**	ATCAGTCATCCCGAAGAGGA	AGCTGCCCACTCACCTCAT
**Pou5f1**	CAAGTTGGCGTGGAGACTTT	TTGGTTCCACCTTCTCCAAC
**Pax7**	TGTTTTGCTTGCATTCGTTC	CGAATTAGACAGAGGCGAAGA
**Pde1c**	CAAAGCCAAAGAAGCTACCG	GCTGCTCAGGCTCATCCTAC
**Six1**	GAGCGAAGGGTGAAACTTCC	TTAAGAACCGGAGGCAAAGA
**Snai1**	CACCCTCATCTGGGACTCTC	GAGCTTTTGCCACTGTCCTC
**St8sia4**	CCGTAGCAGGGAAACGATAA	ACCGAGCTCACAACGACTCT
**Tgm2**	TCCACTCTTCTCAGCCCTGT	TGTTCTCCAAGCCCAGATTC
**Tnip2**	CCCTCATAACCCAGCTCCTT	GTCGCGAGGATATGAAGCTC
**Zyx**	CGCTTAGACCGAGGAGTGTC	GAGACGGAGACGGAGATCG
**Genomic control**	ATTTTGTGCTGCATAACCTCCT	TAGCAACATCCTAAGCTGGACA
