# Supplementary material for: Genomewide Analysis of PRC1 and PRC2 Occupancy Identifies Two Classes of Bivalent Domains
Source: PLoS Genet. 2008 Oct 31;4(10):e1000242. doi: 10.1371/journal.pgen.1000242 (PMC2567431; doi:10.1371/journal.pgen.1000242)
Supplement: Text S1 — Supporting information on the specificity of antibodies. Western blots using mouse ES cell protein extracts demonstrate the specificity of anti-Ring1B and anti-Ezh2 (Active Motif 39103), antibodies used in this study. *Indicates the expected molecular weight. Previous publications that demonstrate the specificity of the antibodies used are listed. (1.83 MB PDF) [file pgen.1000242.s015.pdf]

Text S1 Supporting information on the specificity of antibodies

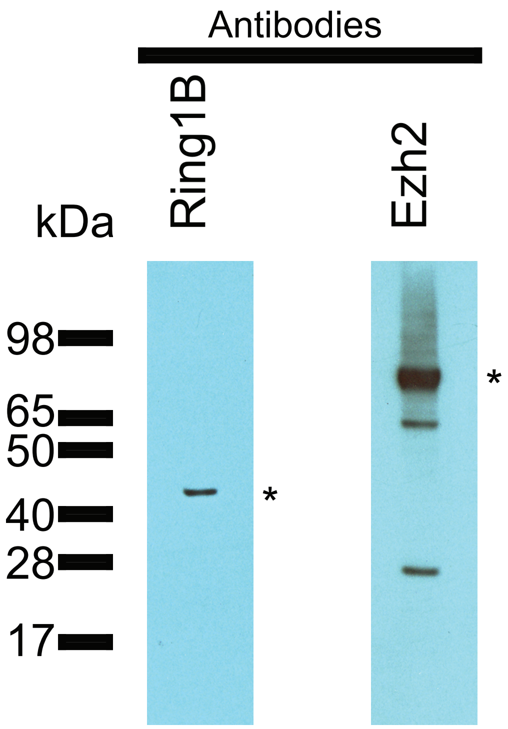

Western blots using mouse ES cell protein extracts demonstrate the specificity of anti-Ring1B (see below) and anti-Ezh2 (Active Motif 39103), antibodies used in this study. *indicates the expected molecular weight. Previous publications that demonstrate the specificity of the antibodies used are listed below.

Anti-H3K4me3 (Abcam ab8580):
Santos-Rosa H, Schneider R, Bannister AJ, Sherriff J, Bernstein BE, Emre NC, Schreiber SL, Mellor J, Kouzarides T. Active genes are trimethylated at K4 of histone H3. Nature 419:407-11 (2002).

Anti-H3K27me3 (Upstate 07-449):
Peters AH, Kubicek S, Mechtler K, O'sullivan RJ, Derijck AA, Perez-Burgos L, Kohlmaier A, Opravil S, Tachibana M, Shinkai Y, Martens JH, Jenuwein T. Partitioning and plasticity of repressive histone methylation states in mammalian chromatin. Mol Cell. Dec;12(6): 1577-89. (2003).

Anti-H3K36me3 (Abcam ab9050):
Lee MG, Norman J, Shilatifard A, Shiekhattar R. Physical and Functional Association of a Trimethyl H3K4 Demethylase and Ring6a/MBLR, a Polycomb-like Protein. Cell 128: 877-87 (2007).

Anti-Suz12 (Abcam ab12073):
Pasini D, Hansen KH, Christensen J, Agger K, Cloos PA, Helin K. Coordinated regulation of transcriptional repression by the RBP2 H3K4 demethylase and Polycomb-Repressive Complex 2. Genes Dev 22:1345-55 (2008).

Anti-Ring1B:
Atsuta T, Fujimura S, Moriya H, Vidal M, Akasaka T, Koseki H. Production of monoclonal antibodies against mammalian Ring1B proteins. Hybridoma 20:43-46. (2001).
