# Supplementary material for: Genomewide Analysis of PRC1 and PRC2 Occupancy Identifies Two Classes of Bivalent Domains
Source: PLoS Genet. 2008 Oct 31;4(10):e1000242. doi: 10.1371/journal.pgen.1000242 (PMC2567431; doi:10.1371/journal.pgen.1000242)
Supplement: Text S2 — Relevant references for transcription factors (TFs) that correspond to implicated motifs and are active in ES cells. (0.63 MB PDF) [file pgen.1000242.s016.pdf]

Text S2 Relevant references for transcription factors (TFs) that correspond to implicated motifs and are active in ES cells.

TFs associated with Ezh2-positive CpG islands

NRSF

Chen ZF, Paquette AJ, Anderson DJ. NRSF/REST is required in vivo for repression of multiple neuronal target genes during embryogenesis. Nat Genet. 1998 Oct;20(2):136-42.
Naruse Y, Aoki T, Kojima T, Mori N. Neural restrictive silencer factor recruits mSin3 and histone deacetylase complex to repress neuron-specific target genes. Proc Natl Acad Sci U S A. 1999 Nov 23;96(24):13691-6.
Huang Y, Myers SJ, Dingledine R. Transcriptional repression by REST: recruitment of Sin3A and histone deacetylase to neuronal genes. Nat Neurosci. 1999 Oct;2(10):867-72.
Lunyak VV, Burgess R, Prefontaine GG, Nelson C, Sze SH, Chenoweth J, Schwartz P, Pevzner PA, Glass C, Mandel G, Rosenfeld MG. Corepressor-dependent silencing of chromosomal regions encoding neuronal genes. Science. 2002 Nov 29;298(5599):1747-52. Epub 2002 Oct 24. Erratum in: Science. 2003 Mar 14;299(5613):1663.
Su X, Kameoka S, Lentz S, Majumder S. Activation of REST/NRSF target genes in neural stem cells is sufficient to cause neuronal differentiation. Mol Cell Biol. 2004 Sep;24(18):8018-25.
Murai K, Naruse Y, Shaul Y, Agata Y, Mori N. Direct interaction of NRSF with TBP: chromatin reorganization and core promoter repression for neuron-specific gene transcription. Nucleic Acids Res. 2004 Jun 14;32(10):3180-9.
Singh SK, Kagalwala MN, Parker-Thornburg J, Adams H, Majumder S. REST maintains self-renewal and pluripotency of embryonic stem cells. Nature. 2008 May 8;453(7192):223-7. Epub 2008 Mar 23.

Cux1

Mailly F, Bérubé G, Harada R, Mao PL, Phillips S, Nepveu A. The human cut homeodomain protein can repress gene expression by two distinct mechanisms: active repression and competition for binding site occupancy. Mol Cell Biol. 1996 Oct;16(10):5346-57.
Lizarraga G, Lichtler A, Upholt WB, Kosher RA. Studies on the role of Cux1 in regulation of the onset of joint formation in the developing limb. Dev Biol. 2002 Mar 1;243(1):44-54.
Truscott M, Harada R, Vadnais C, Robert F, Nepveu A. p110 CUX1 cooperates with E2F transcription factors in the transcriptional activation of cell cycle-regulated genes. Mol Cell Biol. 2008 May;28(10):3127-38. Epub 2008 Mar 17.
Harada R, Vadnais C, Sansregret L, Leduy L, Bérubé G, Robert F, Nepveu A. Genome-wide location analysis and expression studies reveal a role for p110 CUX1 in the activation of DNA replication genes. Nucleic Acids Res. 2008 Jan;36(1):189-202. Epub 2007 Nov 14.

TFs associated with Ezh2-negative CpG islands

Ets family (Ets1, Ets2)

Bhat, N. K.; Thompson, C. B.; Lindsten, T.; June, C. H.; Fujiwara, S.; Koizumi, S.; Fisher, R. J.; Papas, T. S. Reciprocal expression of human ETS1 and ETS2 genes during T-cell activation: regulatory role for the protooncogene ETS1. Proc. Nat. Acad. Sci. 1990 87: 3723-3727.

Ohtani, N.; Zebedee, Z.; Huot, T. J. G.; Stinson, J. A.; Sugimoto, M.; Ohashi, Y.; Sharrocks, A. D.; Peters, G.; Hara, E. Opposing effects of Ets and Id proteins on p16(INK4A) expression during cellular senescence. Nature 2001 409: 1067-1070.

Wen, F.; Tynan, J. A.; Cecena, G.; Williams, R.; Munera, J.; Mavrothalassitis, G.; Oshima, R. G. Ets2 is required for trophoblast stem cell self-renewal. Dev. Biol. 2007 312: 284-299.

Oikawa, T., and Yamada, T. Molecular biology of the Ets family of transcription factors. Gene 2001 303, 11–34.

Sumanas, S., and Lin, S. Ets1-related protein is a key regulator of vasculogenesis in zebrafish. PLoS Biol. 2006 4, e10. 10.1371

Hollenhorst, P. C.; Shah, A. A.; Hopkins, C.; Graves, B. J. Genome-wide analyses reveal properties of redundant and specific

Gabpa (NRF2)

LaMarco, K., Thompson, C. C., Byers, B. P., Walton, E. M. & McKnight, S. L. Identification of Etsand notch-related subunits in GA binding protein. Science 1991 253, 789–792.

Gugneja, S.; Virbasius, J. V.; Scarpulla, R. C. Four structurally distinct, non-DNA-binding subunits of human nuclear respiratory factor 2 share a conserved transcriptional activationdomain. Mol. Cell. Biol. 1995 15: 102-111.

Ristevski S, O'Leary DA, Thornell AP, Owen MJ, Kola I, Hertzog PJ. The Ets transcription factor GABPalpha is essential for early embryogenesis. Mol. Cell Biol. 2004 24, 5844-5849

Resendes, K. K. & Rosmarin, A. G. Interaction between GABPalpha pointed domain and p300 mediates retinoic acid induced transcriptional activation of CD18 in myeloid cells. Mol. Cell Biol. 2006 26, 3060–3070

Yang ZF, Mott S, Rosmarin AG. The Ets transcription factor GABP is required for cell-cycle progression. Nature Cell Biology 2007 9, 339–346.

YY1 (closely related to Rex1)

Park, K.; Atchison, M. L. Isolation of a candidate repressor/activator, NF-E1 (YY-1, delta), that binds to the immunoglobulin kappa 3-prime enhancer and the immunoglobulin heavy-chain micro-E1 site. Proc. Nat. Acad. Sci. 1991 88: 9804-9808.

Hariharan N., Kelley D.E. and Perry R.P., Delta, a transcription factor that binds to downstream elements in several polymerase II promoters, is a functionally versatile zinc fingerprotein, Proc. Natl. Acad. Sci. USA 1991 88 pp. 9799–9803

Wilkinson, F. H., Park, K. & Atchison, M. L. Polycomb recruitment to DNA in vivo by the YY1 REPO domain. Proc. Natl Acad. Sci. 2006 103, 19296–19301

Cunningham, J. T.; Rodgers, J. T.; Arlow, D. H.; Vazquez, F.; Mootha, V. K.; Puigserver, P. mTOR controls mitochondrial oxidative function through a YY1-PGC-1-alpha transcriptional complex. Nature 2007 450: 736-740.

Kim J., Chu J., Shen X., Wang J., Orkin S. An extended transcriptional network for pluripotency of embryonic stem cells. Cell 2008 132, 1049–1061.

NFY family

Li Q, Herrler M, Landsberger N, Kaludov N, Ogryzko VV, Nakatani Y, Wolffe AP. Xenopus NF-Y pre-sets chromatin to potentiate p300 and acetylation-responsive transcription from the Xenopus hsp70 promoter in vivo. EMBO J 1998 17(21): 6300-15.

Faniello MC, Bevilacqua MA, Condorelli G, de Crombrugghe B, Maity SN, Avvedimento VE, Cimino F, Costanzo F. (1999). The B subunit of the CAAT-binding factor NFY binds the central segment of the Co-activator p300. J Biol Chem 274(12): 7623-6.

Mantovani, R., 1999. The molecular biology of the CCAAT-binding factor NFY. Gene 239, 15–27.

Gilthorpe J, Vandromme M, Brend T, Gutman A, Summerbell D, Totty N, Rigby PW. Spatially specific expression of Hoxb4 is dependent on the ubiquitous transcription factor NFY. Development 2002 129(16): 3887-99.

Frontini M, Imbriano C, diSilvio A, Bell B, Bogni A, Romier C, Moras D, Tora L, Davidson I, Mantovani R. NF-Y recruitment of TFIID, multiple interactions with histone fold TAF(II)s. J Biol Chem 2002 277(8): 5841-8.

Udayakumar TS, Belakavadi M, Choi KH, Pandey PK, Fondell JD. Regulation of Aurora-A kinase gene expression via GABP recruitment of TRAP220/MED1. J Biol Chem 2006 281(21): 14691-9.

Donati G, Gatta R, Dolfini D, Fossati A, Ceribelli M, Mantovani R. An NF-Y-dependent switch of positive and negative histone methyl marks on CCAAT promoters. PLoS ONE 2008 3(4): e2066.

Atf family

Hai, T.; Liu, F.; Coukos, W. J.; Green, M. R. Transcription factor ATF cDNA clones: an extensive family of leucine zipper proteins able to selectively form DNA-binding heterodimers. Genes Dev. 1989 3: 2083-2090.

Liu, F. & Green, M. A specific member of the ATF transcription factor family can mediate transcription activation by the adenovirus E1A protein. Cell 1990 61, 1217–1224.

Li M, Baumeister P, Roy B, Phan T, Foti D, Luo S, Lee AS. ATF6 as a transcription activator of the endoplasmic reticulum stress element: thapsigargin stress-induced changes and synergistic interactions with NF-Y and YY1. Mol Cell Biol 2000. 20(14): 5096-106.

Kawasaki, H., L. Schiltz, Chiu R, Itakura K, Taira K, Nakatani Y, Yokoyama KK.ATF-2 has intrinsic histone acetyltransferase activity which is modulated by phosphorylation. Nature 2000 405(6783):195-200.

Yoshida, H., Matsui, T., Yamamoto, A., Okada, T. and Mori, K. XBP1 mRNA is induced by ATF6 and spliced by IRE1 in response to ER stress to produce a highly active transcription factor. Cell 2001 107, pp. 881–891.

Myc family

Gomez-Roman, N., C. Grandori, Eisenman RN, White RJ. Direct activation of RNA polymerase III transcription by c-Myc. Nature 2003 421(6920): 290-4.

Grandori, C., N. Gomez-Roman, Felton-Edkins ZA, Ngouenet C, Galloway DA, Eisenman RN, White RJ. c-Myc binds to human ribosomal DNA and stimulates transcription of rRNA genes by RNA polymerase I. Nat Cell Biol 2005. 7(3): 311-8.

Lin JM, Collins PJ, Trinklein ND, Fu Y, Xi H, Myers RM, Weng Z. Transcription factor binding and modified histones in human bidirectional promoters. Genome Res. 2007 Jun;17(6):818-27.

Takahashi K, Yamanaka S. Induction of pluripotent stem cells from mouse embryonic and adult

fibroblast cultures by defined factors. Cell. 2006 Aug 25;126(4):663-76. Epub 2006 Aug 10.
Kim J, Chu J, Shen X, Wang J, Orkin SH. 2008 An extended transcriptional network for pluripotency of embryonic stem cells. Cell 2008 132(6): 1049-61.
